# Digital protection scheme based on Durbin Watson and Pearson similarity indices for current signals practically applied to power transformers

**DOI:** 10.1038/s41598-025-91491-1

**Published:** 2025-04-10

**Authors:** R. A. Mahmoud

**Affiliations:** Department of Electrical Power and Machines Engineering (PME), College of Engineering Science & Technology, Misr University for Science and Technology (MUST), 6th of October City, Giza Egypt

**Keywords:** Power transformers, Series faults, Inter-turn faults, Shunt faults, Durbin-Watson function, Correlation function, Imbalance coefficients, Energy science and technology, Engineering

## Abstract

Electrical faults can change the power quality parameters of power systems. A numerical protection technique for fault detection and imbalance assessment based the Durbin-Watson (DW) factors for three phase currents is proposed in this paper. The approach integrates two protection functions based on the Durbin-Watson and Pearson similarity algorithms into one protection scheme. This strategy can figure out online faults located on the three-phase power transformer windings, such as turn-to-turn, winding-to-neutral, and winding-to-winding. Moreover, it can distinguish between balanced and imbalanced currents. To assess the validity of the protection scheme, it is practically examined on a three-phase power transformer with tapped windings connected to a three-phase load. Comprehensive tests are conducted to investigate the efficacy and efficiency of the suggested scheme. The analog-to-digital converter is integrated with LABVIEW software to process and analyze the two algorithms of the suggested scheme. The results of the experiments reveal that the security, dependability and precision ratios of the developed protection are almost 99%. Additionally, the protection system can immediately identify electrical faults, triggering a tripping signal to both the annunciator panel and the circuit breaker trip coil of the equipment, but it remains inactive under normal operating conditions and acceptable current unbalance. In the fault events, the numerical approach can respond quickly using a limited data set within a single cycle of the foundation frequency, and operate effectively using a pair of algorithms based on DW and Pearson similarity. It is also robust against the condition of sound transformer windings. Besides, it can determine and estimate the severity rate of perturbation and unbalance in power transformer currents, and it has a protection redundancy. Furthermore, the scheme is extremely sensitive to light fault currents, and has a unique set of tripping curves.

## Introduction

A power transformer is one of the most critical components in electrical power systems. It is widespread in the generation, transmission and distribution systems. Electrical faults on its windings may happen during its operation as a result of environmental or human circumstances. Massive damage to the power transformer may affect the stability of power systems. Therefore, it is imperative to ensure a stable and reliable operation of the transformer in order to sustain a power supply and avert a financial loss for utility companies^1^. Common causes of failure and unexpected shutdown of the transformer are turn-to‐turn, coil-to-neutral, and coil-to-coil faults^2^. The majority of studies have demonstrated that the onset of windings degradation is responsible for 70.0 to 80.0% of transformer failures^3^. As a consequence, online monitoring methods and early fault detection are necessary to avoid unforeseen incidents and minimize the harms related to its operation^4^. A variety of monitoring strategies were employed to diagnose faults in power transformers of different sizes. Digital protection relays/systems are the most efficient and effective tools used to identify transformer winding faults^5^. Numerous techniques were developed to detect turn-to‐turn faults on the transformer windings, particularly those with high voltage. In addition, other protection systems were introduced to efficiently find and distinguish among different faults, like winding-to-neutral and winding-to-winding faults. Differential current protective relays resemble the main protection for the power transformer. These relays are block in the presence of second harmonic components resulting from inrush currents, as well as the fifth harmonic components due to over-fluxing situations, in order to inhibit incorrect tripping of transformer circuit breakers^6,7^.

Numerical differential current relays based on current phasors are the most common solution for protecting transformer windings^1^. These devices could be equipped with additional effective mechanisms to accurately differentiate between internal faults and inrush currents. Harmonics analysis is one mechanism that could be used to estimate the percentage of the second harmonic component relative to the fundamental current component in the differential current^2^. Furthermore, faults between turns are considered to be a critical problem for the differential current relays because they reduce the magnitude of short-circuit current that blinds these relays. Besides, grounding faults located near the neutral point of the three-phase windings of the transformer may make the relay fail to detect these types of faults^3^. This scenario arises when the neutral is grounded through high fault resistance. This drawback can be addressed utilizing restricted earth fault relays^4^, which have more sensitivity than the differential current relays^3,4^. Nevertheless, they fail in certain fault scenarios that occur near the neutral point or from turn to turn. Hence, the sensitive earth fault relay serves as a secondary protection for the differential current relay, necessitating further considerations for current transformers. In^5^, the negative-symmetrical components of the voltage and current measurements were used to find the faults between turns. The superimposed quantities were used to the negative- and positive-sequence components of the differential currents in order to detect the faults between windings/turns in^6^. However, the lack of negative-sequence components makes the method unreliable for three-phase symmetrical faults^6^. In^7^, a transformer winding displacement and axial deformation were applied to identify internal faults.

In recent years, machine learning approaches were utilized to monitor and diagnose faults, like Improved Artificial Neural Networks^8,9^, Vague Support Vector Machines^10^, Support Vector Machines^11,12^, Parzen window^13^, Bayesian networks^14^, and Deep Learning^15^. In^16^, inter-turn and shunt faults were determined using the Second Central Moment (SCM). In addition, various methods based on processing digital signals and mathematical transformations were developed. A mathematical morphology was applied in^17^ to detect internal faults utilizing differential current wave characteristics. In order to obtain the most pertinent information from differential currents using diverse frequency bands, the Wavelet Transform was presented^18,19^. Nevertheless, the substantial computation involved in the Wavelet Transform approaches was a drawback. In^20^, the Empirical Fourier Transform (EFT) was used to distinguish between internal and inter-turn faults by inspecting the directional flow of unilateral and bilateral currents, as well as transformer inrush currents. But the approach was not examined in the instance of over-excitation and recuperation/sympathetic type of magnetizing inrush state^20^. In^21^, the authors presented a computational technique to discriminate between faults and inrush currents using the parameter values of the power transformer. In^22^, short-circuit impedance, sweep impedance, and frequency response were used to identify a transformer winding deformation. This approach could provide an approximate location of the fault, but it was unable to assess the level of the damage^22^. In^23^, a differential protection was contingent on the Hausdorff distance (HD) criterion to differentiate between internal faults, magnetizing inrush currents and external faults accompanied with CT saturation extent for the transformer. In^24^, a high-order statistic based on the behavior of the kurtosis index was used to find and distinguish internal faults. Even so, the discrimination limit was dependent on the power system arrangement. In^25^, a Kalman-filter technique was applied to estimate the primary current of the transformer windings, and the estimation error was able to distinguish internal faults from transient currents. However, the approach used the parameter values of the transformer, and it was only investigated on single-phase power transformers^25^. The hysteresis curves were used to detect inter-turn faults in the power transformer windings^26^. However, the method used the information gathered from the obtainable saturation charts directly to figure out the faults. In^27^, the approach was developed to detect the leakage flux distribution during inter-turn faults using diverse concentric rectangular spiral windings. Although the method was able to find the inter-turn faults, it was unable to identify the inter-turn faults at the winding center-point^27^. Additionally, the transformer design needs to be changed to accommodate the sensors’ installation^27^. In^28^, computational techniques were applied to classify mechanical and electrical faults. A sweep frequency impedance-based method was used to identify and assess the severity of turn-to-turn faults within power transformer windings^29^. In^30^, a proposed index was used for the Frequency Response Analysis (FRA) of the transformer with mechanical faults. A method based on different indices was presented to classify faults in^31^. In^32^, the FRA and disk-to-disk methods were used to identify and locate inter-turn faults in the transformer windings. In^32^, the fault detection method did not examine the situation of three-phase windings faults with an iron core. A gas chromatography test (i.e., dissolved gas analysis) of the transformer oil was conducted to observe the transformer fault conditions^33^. In^34^, turn-to-turn faults were identified using a transformer leakage flux analysis based on Vibration‐Based Method (VBM), Flux Leakage‐Based Method (FLBM), and Search Coil‐Based Method (SCBM). In^35^, negative-sequence component and space-vector algorithms was compared to find transformer turn-to-turn faults. In^36^, the differences in both positive-sequence impedance and negative-sequence current of the transformer were used to sense the inter-turn faults. The relay settings were derived from transformer nameplate data and test reports^36^.

In this article, the authors suggest a novel protection strategy to detect fault currents and assess imbalance events using both Durbin-Watson (DW) and Pearson similarity functions. This strategy is experimentally examined on a three-phase power transformer with ten taps for each phase winding. The quantitative findings of both protection functions will be compared to measure their performance when detecting transformer faults and evaluating currents asymmetry. The testing outcomes assure that the proposal possesses the following qualities:

1. The protection scheme can be used to define steady-state conditions, and faults situated on the three-phase windings of the power transformer. These faults may be series, shunt, or turn-to-turn faults,
2. The trajectories of Durbin-Watson and correlation factors estimated for current signals can be continuously monitored, and the instances of shunt and turn-to-turn faults can be promptly identified,
3. The quantity of Durbin-Watson is negligible in steady-state conditions, but its value is considerable in fault events, including turn-to-turn, winding-to-neutral, and winding-to-winding faults,
4. The technique can be used to determine the difference between unbalanced and balanced currents,
5. The Durbin-Watson statistic is a loss function that can be sensitive to class imbalances and fault conditions,
6. The protection approach only uses local current measurements, which reduces the time it takes to send and process data,
7. The developed method can be applied in smart systems with a wide range of voltage and power ratings,
8. Dual protection functions can be combined to establish a multi-function digital protection scheme, which includes current phase balance and phase overcurrent protections,
9. The protection scheme can be practically exploited,
10. The approach can be employed to protect the single-phase or three-phase windings of AC machines,
11. The advanced algorithms can be described as intelligent, reliable, fast, and precise protection,
12. The scheme is active when a fault occurs, but it is not active when the machine windings are sound,
13. The developed scheme combines two numerical techniques in order to increase the reliability and redundancy of the protection,
14. The protection settings for the two algorithms can be selected without the need for any mathematical calculations,
15. It is possible to modify the fault detection sensitivity and speed using the data set and the predetermined settings of the Durbin-Watson and correlation algorithms,
16. The method can function online, and it can also be incorporated with other microprocessors-based protection techniques,
17. The approach has the ability to evaluate the intensity of perturbation and asymmetry in three-phase currents,
18. The methodology can protect power transformers with different power and voltage specifications,
19. The selected settings do not change with variations in power transformer parameters, size, or design,
20. The developed methodology exhibits stability despite varying the specifications of power transformer parameters, and
21. The tripping time of the protection scheme can be estimated using new mathematical expressions based on the Durbin-Watson and auto-correlation factors, where this time corresponds to the level of short-circuit current.

This article is organized in the following sequence: The mathematical models and the steps of the protection algorithms are elaborately detailed in "Proposed scheme". A laboratory system under test is described in "Laboratory system under test" for investigating the performance of the advanced protection scheme. The practical results are analyzed and explained in "Experiments and statistical analysis". In "Protection scheme features", the effectiveness and benefits of the protection scheme are demonstrated, along with a thorough comparison between the present and previous techniques. The main contributions are enumerated in "Contributions". In conclusion, the laboratory outcomes of the examination are summarized in "Conclusions".

## Proposed scheme

The suggested protection scheme integrates two computational techniques, encompassing the Durbin-Watson and the correlation techniques.

### Mathematical models

#### Durbin-Watson model

The Durbin-Watson (DW) factor is a test applied to identify serial correlation in the residuals from a statistical model or regression analysis^37^. In other sense, the Durbin-Watson test assesses whether there is auto-correlation among the residuals of time series data or not. The Durbin-Watson factor (*DW*) has the following properties^37^:

1. Durbin-Watson factor is a dimensionless quantity,
2. The Durbin-Watson statistic is a test for auto-correlation in a regression model’s output,
3. When the DW factor is 2.0, there is a zero serial correlation detected in the measurements. When its value is below 2.0, there is a positive serial correlation, but if its value is above 2.0, there is a negative serial correlation,
4. When the relationship between any two data sets is linear, the DW quantity is close to zero,
5. It can be useful in technical analysis and utilized for comparing diverse physical quantities,
6. It is highly sensitive to non-linear values of any measurable variable,
7. The measurement scale of the variable has no impact on the Durbin-Watson factor, and
8. From a signal processing perspective, the DW estimator performs the functional role of a digital low-pass filter because a smoothing can be accomplished with a data set concept. Thus, the variance from short-term variations can be reduced by the DW estimator.

In this study, the following assumptions regarding the test should be taken into account:

1. Estimated errors are typically distributed with a mean value of zero, and
2. All estimated errors remain constant.

The Durbin-Watson factor (*DWi*_*s*_) can be estimated between each of the two data sets differing by a single cycle, where each data set contains *N*_*w*_ samples of the measurements of an electrical current signal (*i*_*s*_(*k*)) taken for each *S* phase, where *S* stands for phase *A*, *B* or *C*.

The Durbin-Watson factor (*DWi*_*s*_) can be expressed as follows^37^:

1 $$DWi_{s} = \frac{{\sum\limits_{k = 1}^{{N_{w} }} {[i_{s} (k) - i_{s} (k - N_{s} )} ]^{2} }}{{\sum\limits_{k = 1}^{{N_{w} }} {[i_{s} (k)} ]^{2} }}$$

Also, the Durbin-Watson factor ((d*DWi*_*s1*_)) can be defined as follows^37^:

2 $$dDWi_{s1} = \frac{{\sum\limits_{k = 1}^{{N_{w} }} {[i_{s} (k + N_{s} ) - i_{s} (k)} ]^{2} }}{{\sum\limits_{k = 1}^{{N_{w} }} {[i_{s} (k + N_{s} )} ]^{2} }}$$

The mathematical formula (1) can be used to quantify the following three DW factors: *DWi*_*a*_, *DWi*_*b*_*,* and *DWi*_*c*_*.* Also*,* the mathematical Eq. (2) can be used to calculate the following three DW factors: *dDWi*_*a1*_, *dDWi*_*b1*_*,* and* dDWi*_*c1*_. In consequence, six DW factors can be used in the suggested protection scheme to decide the appropriate action, as given below.

1. A tripping signal is sent to the circuit breakers(s) of the protected power transformer in the instance of a fault or an unacceptable current imbalance. The majority of electrical fault events for the power transformers are due to turn-to-turn, winding-to-neutral, and winding-to-winding faults.
2. A restraining signal is issued to the circuit breakers(s) of the protected power transformer in the case of a normal operation or an acceptable current imbalance.

In this work, the DW factors can be used to detect various faults and compare the balance and imbalance of three-phase currents. Testing results on a power transformer with tapped windings will prove that the DW instrument can effectively and efficiently identify transformer faults, making the developed technique is extremely accurate and reliable.

#### Correlation model

##### Cross-correlation model

Consider a series data of *N*_*w*_ per each data packages of two phase current signals (*i*_*s*_ and *i*_*x*_) indexed by *k* 1, 2,…, *N*_*w*_*,* the cross-correlation coefficient (*ri*_*sx*_) between the two phase currents, namely (*i*_*s*_(*k*) and *i*_*x*_(*k*)), can be formulated as follows^38^:

3 $$ri_{sx} = \frac{{[\sum\limits_{k = 1}^{{N_{w} }} {i_{s} (k)i_{x} (k)} - \frac{1}{{N_{w} }}i_{s} (k)\sum\limits_{k = 1}^{{N_{w} }} {i_{x} (k)} ]}}{{[\sqrt {\sum\limits_{k = 1}^{{N_{w} }} {(i_{s} (k)} )^{2} - \frac{1}{{N_{w} }}(\sum\limits_{k = 1}^{{N_{w} }} {i_{s} (k)} )^{2} } ] \times [\sqrt {\sum\limits_{k = 1}^{{N_{w} }} {(i_{x} (k))^{2} - \frac{1}{{N_{w} }}(\sum\limits_{k = 1}^{{N_{w} }} {i_{x} (k))^{2} ]} } } }}$$

where, *ri*_*sx*_: The cross-correlation coefficient estimated between two corresponding data packages for the two phase currents (*i*_*s*_(*k*) and* i*_*x*_(*k*)) acquired at the supply side for the *S* and *X* phases of the power transformer windings, *i*_*s*_(*k*): The current measurement at the position (*k*) measured at the supply side for the *S* phase of the power transformer windings, *i*_*x*_(*k*): The current measurement at the position (*k*) measured at the supply side for the *X* phase of the power transformer windings, *N*_*w*_: The number of measurements per a data package for the current signal*,* and *i*_*a*_(*k*),* i*_*b*_(*k*) and *i*_*c*_(*k*): The measurements of the three-phase currents at position ‘*k*’ taken at the three-phase supply ends of the power transformer windings.

##### Auto-correlation model

The auto-correlation coefficient (*ri*_*s*_) between two data packages, namely *i*_*s*_(*k*) and *i*_*s*_(*k-N*_*s*_) can be expressed as given below^39^.

4 $$ri_{s} = \frac{{[\sum\limits_{k = 1}^{{N_{w} }} {i_{s} (k)i_{s} (k - N_{s} )} - \frac{1}{{N_{w} }}i_{s} (k)\sum\limits_{k = 1}^{{N_{w} }} {i_{s} (k - N_{s} )} ]}}{{[\sqrt {\sum\limits_{k = 1}^{{N_{w} }} {(i_{s} (k)} )^{2} - \frac{1}{{N_{w} }}(\sum\limits_{k = 1}^{{N_{w} }} {i_{s} (k)} )^{2} } ] \times [\sqrt {\sum\limits_{k = 1}^{{N_{w} }} {(i_{s} (k - N_{s} ))^{2} - \frac{1}{{N_{w} }}(\sum\limits_{k = 1}^{{N_{w} }} {i_{s} (k - N_{s} ))^{2} ]} } } }}$$

where, *ri*_*s*_: The auto-correlation coefficient computed between two data packages differing by a single cycle (*N*_*s*_) for the same current *i*_*s*_(*k*) taken at the supply terminals of the power transformer windings, *i*_*s*_(*k-N*_*s*_): The measurement of the current (*i*_*s*_) at the position (*k-N*_*s*_), and *N*_*s*_: The number of measurements per a single cycle for each current signal.

#### Unbalance coefficient estimation

A new unbalance coefficient (*UFi*_*1*_) based on Durbin-Watson factors (*DWi*_*a*_, *DWi*_*b*_, and *DWi*_*c*_) can be used to estimate the unbalance of three-phase currents. It is possible to expresses the coefficient (*UFi*_*1*_) as follows:

5 $$UFi_{1} \% = \begin{array}{*{20}c} {} \\ {} \\ \end{array} Max\left\{ {} \right.\left| {} \right.UFi_{a1} \left. {} \right|,\begin{array}{*{20}c} {} \\ {} \\ \end{array} \left| {} \right.UFi_{b1} \left. {} \right|\begin{array}{*{20}c} {} \\ {} \\ \end{array} or\left| {} \right.UFi_{c1} \left. {} \right|\left. {} \right\}$$

6 $$UFi_{1} \% = \begin{array}{*{20}c} {} \\ {} \\ \end{array} Max\left\{ {} \right.\left| {} \right.\frac{{DWi_{a} - DWi_{m} }}{{DWi_{m} }} \times 100\left. {} \right|,\begin{array}{*{20}c} {} \\ {} \\ \end{array} \left| {} \right.\frac{{DWi_{b} - DWi_{m} }}{{DWi_{m} }} \times 100\left. {} \right|\begin{array}{*{20}c} {} \\ {} \\ \end{array} or\left| {} \right.\frac{{DWi_{c} - DWi_{m} }}{{DWi_{m} }} \times 100\left. {} \right|\left. {} \right\}$$

7 $$DWi_{m} = \begin{array}{*{20}c} {} \\ {} \\ \end{array} \frac{{DWi_{a} + DWi_{b} + DWi_{c} }}{3}$$

where, *UFi*_*1*_: The unbalance coefficient (in %) based on the Durbin-Watson factors quantified for the three-phase transformer currents; the coefficient (*UFi*_*1*_) is the maximum value of the three-phase coefficients (*UFi*_*a1*_*, UFi*_*b1*_ or *UFi*_*c1*_) based on the Durbin-Watson factors (*DWi*_*a*_, *DWi*_*b*_, and *DWi*_*c*_), respectively.

Furthermore, another unbalance coefficient (*UFi*_*2*_) based on cross-correlation factors (*ri*_*ab*_, *ri*_*bc*_, and *ri*_*ca*_) can be used to measure the imbalance of three-phase currents. It is possible to expresses the coefficient (*UFi*_*2*_) as follows:

8 $$UFi_{2} \% = \begin{array}{*{20}c} {} \\ {} \\ \end{array} Max\left\{ {} \right.\left| {} \right.UFi_{ab2} \left. {} \right|,\begin{array}{*{20}c} {} \\ {} \\ \end{array} \left| {} \right.UFi_{bc2} \left. {} \right|\begin{array}{*{20}c} {} \\ {} \\ \end{array} or\left| {} \right.UFi_{ca2} \left. {} \right|\left. {} \right\}$$

9 $$UFi_{2} \% = \begin{array}{*{20}c} {} \\ {} \\ \end{array} Max\left\{ {} \right.\left| {} \right.(ri_{ab} + 0.5) \times 100\left. {} \right|,\begin{array}{*{20}c} {} \\ {} \\ \end{array} \left| {} \right.(ri_{bc} + 0.5) \times 100\left. {} \right|\begin{array}{*{20}c} {} \\ {} \\ \end{array} or\left| {} \right.(ri_{ca} + 0.5) \times 100\left. {} \right|\left. {} \right\}$$

where, *UFi*_*2*_: The imbalance coefficient (in %) based on the cross-correlation factors calculated for the three-phase transformer currents; the coefficient (*UFi*_*2*_) is the maximum value of the three-phase coefficients (*UFi*_*ab2*_*, UFi*_*bc2*_*,* or *UFi*_*ca2*_) based on the three cross-correlation estimators (*ri*_*ab*_, *ri*_*bc*_, and *ri*_*ca*_), respectively. The asymmetry severity can be classified into diverse levels, enabling the operation of the cooling systems regularly for the power transformer required to be protected.

#### Relay tripping time

##### Relay tripping time based on Durbin-Watson

**Fig. 1:**
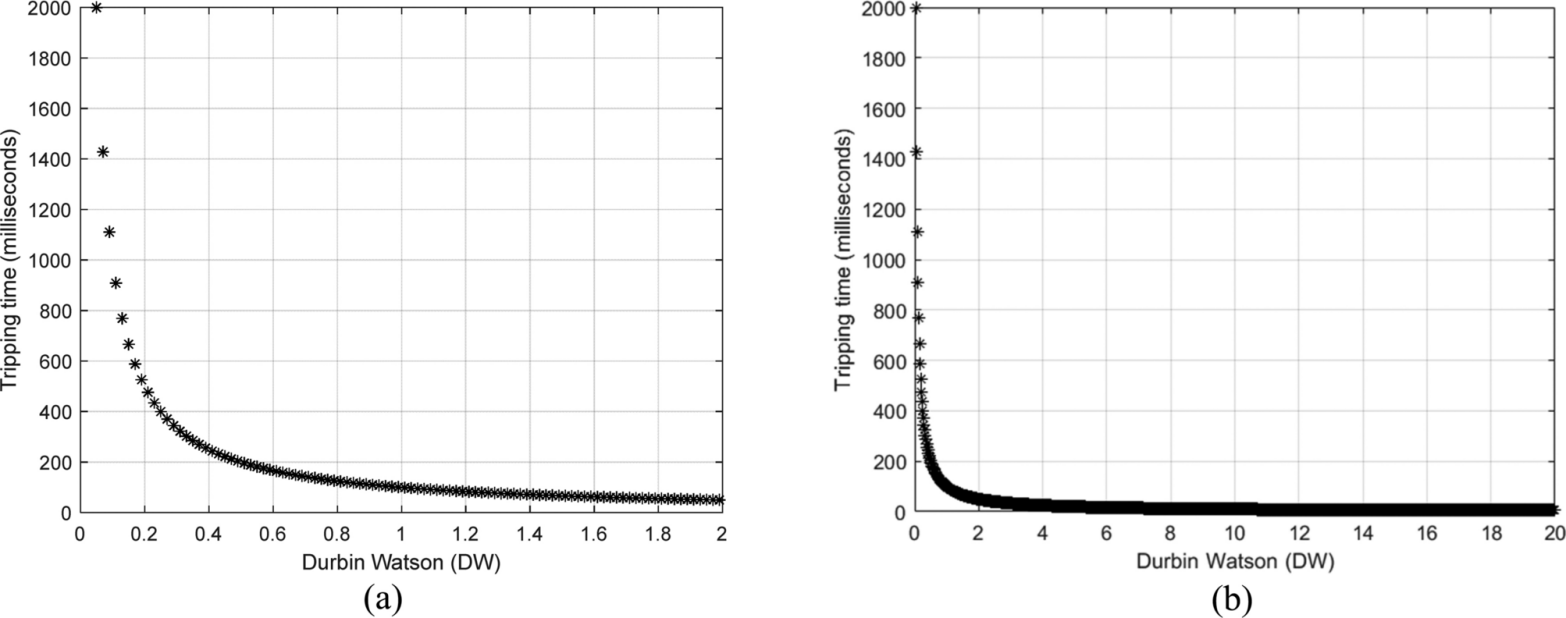
(**a**,**b**) A sample of the DW-tripping time curve for calculating the relay tripping time when *K*_*s1*_ = 1.0 and *DW*_*pu*_ =  + 0.1

It is known that the tripping time of the relay should be infinite when the machine operates normally. In the normal operating conditions, all the *DW* factors of the three-phase currents are nearly zero. Whereas, in fault events, the Durbin-Watson factor of at least one phase current exceeds a prescribed threshold value. With a larger *DW* factor, the tripping time will become shorter, which corresponds to the severity size of the fault current. A new formula of DW-time curves is proposed to quantify the relay tripping time (*T*_*s1*_). The formula needs the Durbin-Watson pickup value (*DW*_*pu*_), the time multiplier (*K*_*s1*_), and the actual Durbin-Watson factor (*DWi*_*s*_) computed for each phase current (*i*_*s*_). The proposed characteristic of the tripping time (*T*_*s1*_) based on the Durbin-Watson factor can be used after identifying the fault, which can be expressed as follows:

10 $$T_{s1} \begin{array}{*{20}c} {} \\ {} \\ \end{array} = \begin{array}{*{20}c} {} \\ {} \\ \end{array} \frac{{K_{s1} }}{{\left| {} \right.(\frac{{\left| {} \right.DWi_{s} \left. {} \right|}}{{DW_{pu} }})^{{}} \left. {} \right|}}$$

11 $$T_{t1} = \begin{array}{*{20}c} {} \\ {} \\ \end{array} Min\left\{ {} \right.\left| {} \right.T_{a1} \left. {} \right|,\begin{array}{*{20}c} {} \\ {} \\ \end{array} \left| {} \right.T_{b1} \left. {} \right|\begin{array}{*{20}c} {} \\ {} \\ \end{array} or\left| {} \right.T_{c1} \left. {} \right|\left. {} \right\}$$

where, *T*_*s1*_: The estimated tripping time (in milliseconds) of the protection using the Durbin-Watson factor (*DWi*_*s*_) for the phase current (*i*_*s*_), *T*_*t1*_: The actual tripping time (in milliseconds) of the relay; it is the lowest value of (*T*_*a1*_*, T*_*b1*_*,* or *T*_*c1*_), *DW*_*pu*_: The selected Durbin-Watson pickup value (the chosen value is *DW*_*pu*_ =  + 0.10), and *K*_*s1*_: The chosen value of time multiplier for the Durbin-Watson algorithm,

Figure 1 depicts a sample of the DW-time curves used to calculate the relay tripping time when *K*_*s1*_ = 1.0 and *DW*_*pu*_ =  + 0.1. Table 1 demonstrates the mechanism used to calculate the three single-phase tripping times (*T*_*a1*_*, T*_*b1*_*,* and *T*_*c1*_) for the current relay based on the three single-phase Durbin-Watson factors (*DWi*_*a*_*, DWi*_*b*_*,* and *DWi*_*c*_), respectively. The actual tripping time of the relay can be the minimum value of (*T*_*a1*_*, T*_*b1*_*,* or *T*_*c1*_). The tripping permission is issued to the power transformer CBs after passing the actual time delay that starts at the moment of the Durbin-Watson pickup.

**Table 1:** The calculated tripping time of the current relay based on the Durbin-Watson coefficient.

Case designation	Durbin-Watson factors range for current relay	Current relay tripping time (in s)	
Case 1; Normal operation	*0.0* ≤ *DWi*_*a*_ ≤ *ΔW,* *0.0* ≤ *DWi*_*b*_ ≤ *ΔW,* and *0.0* ≤ *DWi*_*c*_ ≤ *ΔW*	**∞**	
Case 2: Transformer fault (i.e., turn-to-turn, internal or external shunt fault)	*dDWi*_*a1*_ > *ΔW,* *dDWi*_*b1*_ > *ΔW,* or *dDWi*_*c1*_ > *ΔW*	When* DWi*_*s*_ ≥ *DW*_*pu*_ $$T_{s1} \begin{array}{*{20}c} {} \\ {} \\ \end{array} = \begin{array}{*{20}c} {} \\ {} \\ \end{array} \frac{{K_{s1} }}{{\left| {} \right.(\frac{{\left| {} \right.DWi_{s} \left. {} \right|}}{{DW_{pu} }})^{{}} \left. {} \right|}}\begin{array}{*{20}c} {} & {} & {} \\ \end{array}$$	When* DWi*_*s*_ = *0.0* $$T_{s1} \begin{array}{*{20}c} {} \\ {} \\ \end{array} = \begin{array}{*{20}c} {} \\ {} \\ \end{array} \frac{{K_{s1} }}{{\left| {} \right.(\frac{{\left| {} \right.DWi_{s} \left. {} \right|}}{{DW_{pu} }})^{{}} \left. {} \right|}}\begin{array}{*{20}c} {} \\ {} \\ \end{array} = \alpha \begin{array}{*{20}c} {} & {} & {} \\ \end{array}$$
		1. The protection algorithm will be active when the *DWi*_*a*_*, DWi*_*b*_*,* or *DWi*_*c*_ ≥ *DW*_*pu*_ 2. Estimate the three single-phase tripping times (*T*_*a1*_*, T*_*b1*_*,* and *T*_*c1*_) using the three single-phase Durbin-Watson factors (*DWi*_*a*_*, DWi*_*b*_*,* and *DWi*_*c*_), respectively 3. Select the actual tripping time of the relay, which is the lowest value of the three single-phase tripping times (*T*_*a1*_*, T*_*b1*_*,* or *T*_*c1*_)	

##### Relay tripping time based on correlation

**Fig. 2:**
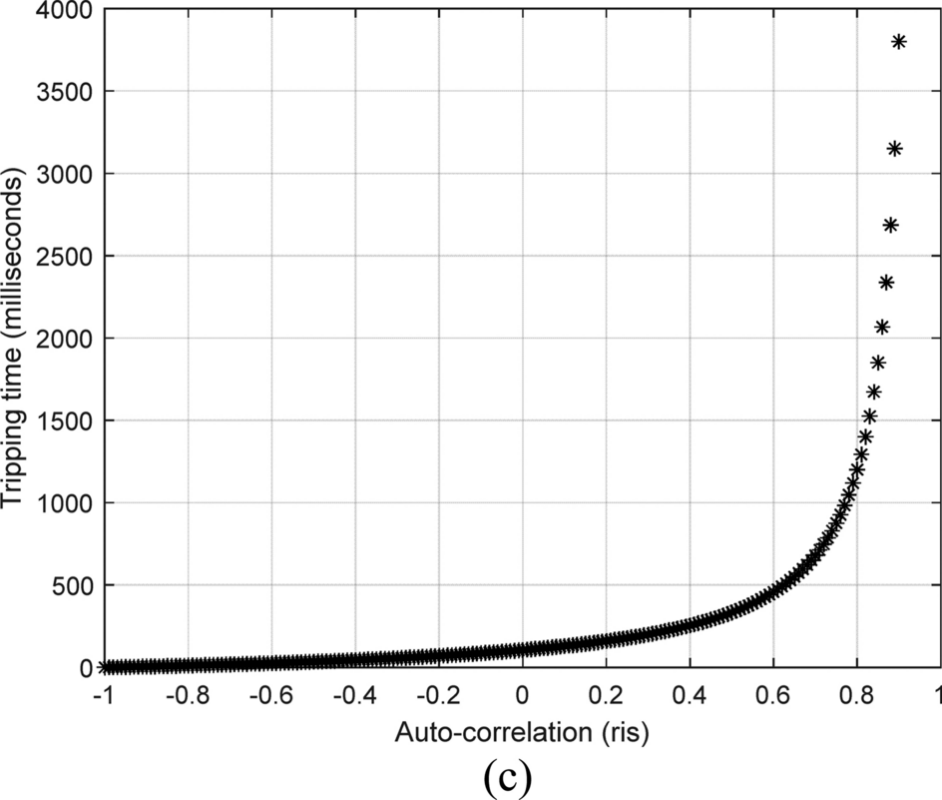
A sample of the auto-correlation-tripping time curve for calculating the relay tripping time when *K*_*s2*_ = 0.1 and *ri*_*pu*_ =  + 0.95.

The tripping time (*T*_*s2*_) can be estimated using another formula in the event of faults. The formula requires the auto-correlation pickup (*ri*_*pu*_), the time multiplier (*K*_*s2*_), and the auto-correlation coefficient *(ri*_*s*_*)* estimate for phase current (*i*_*s*_). The proposed formula used to compute the tripping time (*T*_*s2*_) based on the auto-correlation coefficient is stated below.

12 $$T_{s2} \begin{array}{*{20}c} {} \\ {} \\ \end{array} = \begin{array}{*{20}c} {} \\ {} \\ \end{array} \frac{{K_{s2} }}{{\left| {} \right.(\frac{{1.0 + ri_{pu} }}{{1.0 + ri_{s} }})^{{}} - 1\left. {} \right|}}$$

13 $$T_{t2} = \begin{array}{*{20}c} {} \\ {} \\ \end{array} Min\left\{ {} \right.\left| {} \right.T_{a2} \left. {} \right|,\begin{array}{*{20}c} {} \\ {} \\ \end{array} \left| {} \right.T_{b2} \left. {} \right|\begin{array}{*{20}c} {} \\ {} \\ \end{array} or\left| {} \right.T_{c2} \left. {} \right|\left. {} \right\}$$

where, *T*_*s2*_: The estimated tripping time (in milliseconds) of the protection using the auto-correlation coefficient (*ri*_*s*_) for the phase current (*i*_*s*_), *T*_*t2*_: The actual tripping time (in milliseconds) of the relay; it is the minimum quantity of (*T*_*a2*_*, T*_*b2*_*,* or *T*_*c2*_), *ri*_*pu*_: The prescribed auto-correlation pickup of the relay (it is *ri*_*pu*_ = *1.0 – Δr*_*2*_), *ri*_*s:*_ The actual auto-correlation coefficient quantified for the phase current (*i*_*s*_), and *K*_*s2*_: The chosen value of time multiplier for the auto-correlation algorithm,

The tripping time of the relay is infinite if the power transformer is operating in a regular or normal state, and it follows the mathematical Eq. (12) in the fault events. Figure 2 shows a sample of the auto-correlation-tripping time curve used to estimate the relay tripping time when *K*_*s2*_ = 0.1 and *ri*_*pu*_ =  + 0.95.

### Scheme strategy

To detect fault and asymmetry events, the protection scheme is dependent on two algorithms: the Durbin-Watson algorithm, and the correlation algorithm.

#### Durbin-Watson algorithm procedure

The computational technique based on the Durbin-Watson factors is capable of fulfilling the protection functions of phase overcurrent and unbalanced currents. In order to identify faults and current imbalances, the Durbin-Watson algorithm needs the following:

(I) Three-phase currents, taken using three single-phase current transformers installed at the supply terminals of the protected power transformer, are the input signals to the relay.
(II) Estimation of six Durbin-Watson (DW) factors to determine whether a power transformer state is healthy or faulty, upon which the protection relay issues a tripping permission to the circuit breakers of the defected transformer. However, the relay will remain inactive when the equipment is sound and its currents are balanced.

**Fig. 3:**
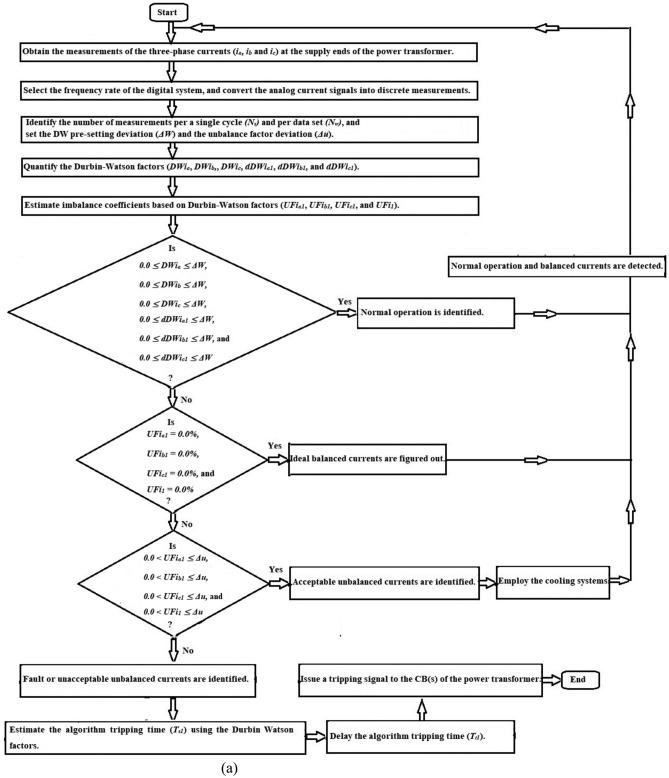
Flow chart of power transformer fault detection and imbalance assessment using the Durbin-Watson and derived unbalance factors.

As shown in Fig. 3, the Durbin-Watson-based algorithm can be executed as follows:

1. Obtain the measurements of the three-phase currents (*i*_*a*_*, i*_*b*_, and *i*_*c*_) using the current transducers constructed at the supply ends of the power transformer,
2. Select the frequency rate of the digital system, then convert the analog current signals into measurement values,
3. Identify the number of measurement values per a single cycle *(N*_*s*_*)*, and per data set *(N*_*w*_*)* as well,
4. Determine the pre-setting deviation of the Durbin-Watson (*ΔW*), and the imbalance factor deviation (*Δu*) as well,
5. Quantify the six Durbin-Watson factors (*DWi*_*a*_, *DWi*_*b*_, *DWi*_*c*_, *dDWi*_*a1*_, *dDWi*_*b1*_*,* and* dDWi*_*c1*_) for the current waves,
6. Estimate the imbalance coefficients (*UFi*_*a1*_, *UFi*_*b1*_*, UFi*_*c1*_, and *UFi*_*1*_) based on Durbin-Watson factors to ensure the presence of out-of-balance, and classify the degree of imbalance into distinct categories,
7. Adopt the proposal rules based on the Durbin-Watson (DW) factors for three-phase phase currents, as included in Table 2,
8. Differentiate between healthy and faulty instances, when the six Durbin-Watson factors are near zero, then the power transformer situation is sound. Otherwise, the equipment state is faulty,
9. Estimate the relay tripping time (*T*_*t1*_) based on the DW factors computed for three-phase currents,
10. Declare a tripping flag to the annunciator panel, and send an isolation signal to the circuit breakers of the power transformer to prevent its failure. It is possible that this is a turn-to-turn, coil-to-neutral or coil-to-coil fault,
11. The data set area (*N*_*w*_) and the DW pre-setting value (*ΔW*) can be used to adjust the sensitivity, security, and response time of the protection strategy,
12. In this algorithm, the DW pre-setting deviation (*ΔW*) should be within the range of 0.0 and + 0.20, with a step value of 0.01. This setting can be adjusted to correspond roughly to the prevailing state of the transformer under protection. The DW pre-setting deviation (*ΔW*) is set to + 0.10.

**Table 2:** The operation rules for Durbin-Watson and correlation algorithms, and the protection action.

Power transformer condition	Correlation algorithm	Durbin-Watson algorithm	Protection action
	The algorithm setting deviations *Δr*_*1*_*, Δr*_*2*_ and* ΔW* are 0.10, 0.05, and 0.10, respectively		
Normal operation	*1.0 – Δr*_*2*_ ≤ *ri*_*a*_ ≤ *1.0,*	*0.0* ≤ *DWi*_*a*_ ≤ *ΔW,*	Healthy condition:
	*1.0 – Δr*_*2*_ ≤ *ri*_*b*_ ≤ *1.0,* and	*0.0* ≤ *DWi*_*b*_ ≤ *ΔW,* and	Blocking action for the power transformer CBs
	*1.0 – Δr*_*2*_ ≤ *ri*_*c*_ ≤ *1.0*	*0.0* ≤ *DWi*_*c*_ ≤ *ΔW*	
Current balance	*-0.5 – Δr*_*1*_ ≤ *ri*_*ab*_ ≤ *-0.5* + *Δr*_*1*_*,*	*0.0* ≤ *dDWi*_*a1*_ ≤ *ΔW,*	Three-phase current balance:
	*-0.5 – Δr*_*1*_ ≤ *ri*_*bc*_ ≤ *-0.5* + *Δr*_*1*_*,* and	*0.0* ≤ *dDWi*_*b1*_ ≤ *ΔW,* and	Blocking action for the power transformer CBs
	*-0.5 – Δr*_*1*_ ≤ *ri*_*ca*_ ≤ *-0.5* + *Δr*_*1*_	*0.0* ≤ *dDWi*_*c1*_ ≤ *ΔW*	
Fault event	*1.0 – Δr*_*2*_ > *ri*_*a*_*,*	*dDWi*_*a1*_ > *ΔW,*	Fault condition (it may be a turn-to-turn, coil-to-neutral or coil-to-coil fault)
	*1.0 – Δr*_*2*_ > *ri*_*b*_*,* or	*dDWi*_*b1*_ > *ΔW,* or	Tripping action for the power transformer CBs
	*1.0 – Δr*_*2*_ > *ri*_*c*_	*dDWi*_*c1*_ > *ΔW*	
Current imbalance	*-0.5 – Δr*_*1*_ > *ri*_*ab*_ > *-0.5* + *Δr*_*1*_	*dDWi*_*a1*_ > *ΔW,*	Three-phase current imbalance
	*-0.5 – Δr*_*1*_ > *ri*_*bc*_ > *-0.5* + *Δr*_*1*_*,* or	*dDWi*_*b1*_ > *ΔW,* or	Tripping action for the power transformer CBs
	*-0.5 – Δr*_*1*_ > *ri*_*ca*_ > *-0.5* + *Δr*_*1*_	*dDWi*_*c1*_ > *ΔW*	

#### Correlation algorithm procedure

The numerical method based on correlation coefficients has the capability to implement the functional roles of phase overcurrent and current unbalance protections. To define the fault and current unbalance states, the correlation algorithm requires the following:

(I) Three-phase current waves, measured using three single-phase current transducers constructed at the supply ends of the power transformer, are the analog input to the relay, and
(II) The algorithm of three cross-correlation and three auto-correlation coefficients serves as a redundant approach to detect abrupt changes in current signals during fault situations. In the event of any fault or unsymmetrical currents, the protection relay initiates a tripping order to the power transformer circuit breakers. Whereas, it will be idle when the transformer is healthy and its currents are symmetrical.

**Fig. 4:**
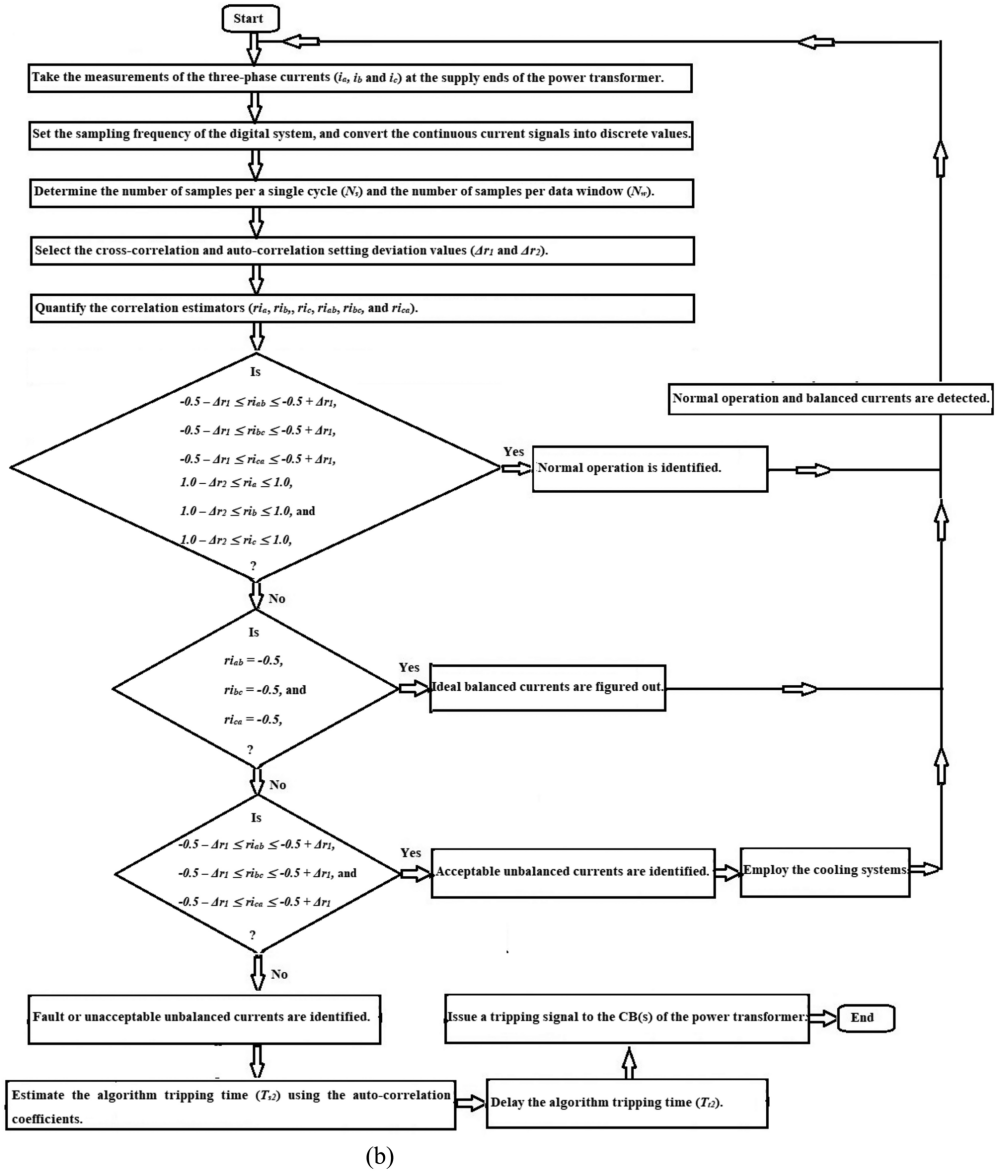
Flow chart of power transformer fault detection and imbalance assessment using the correlation coefficients.

**Fig. 5:**
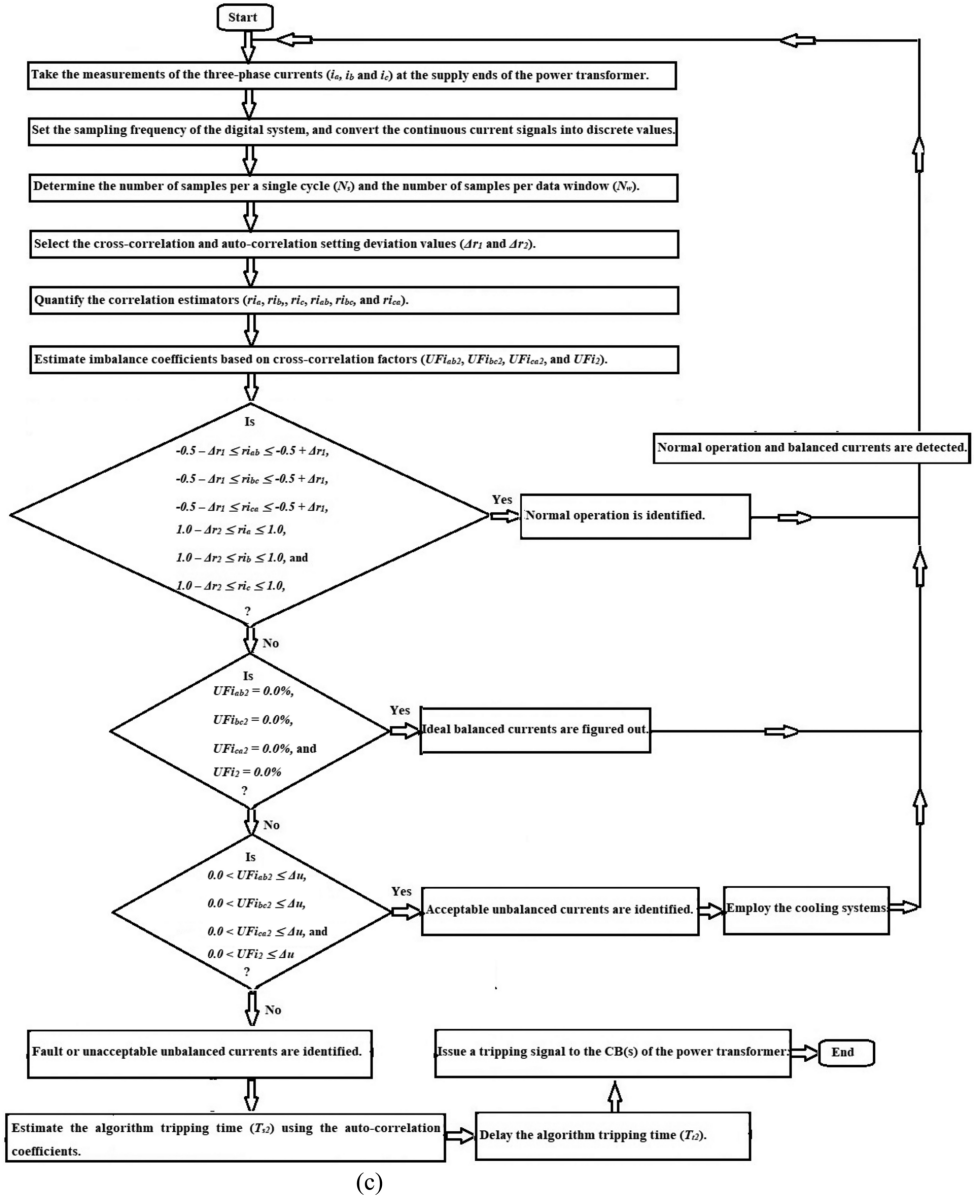
Flow chart of power transformer fault detection and imbalance assessment using the correlation and derived unbalance factors.

**Fig. 6:**
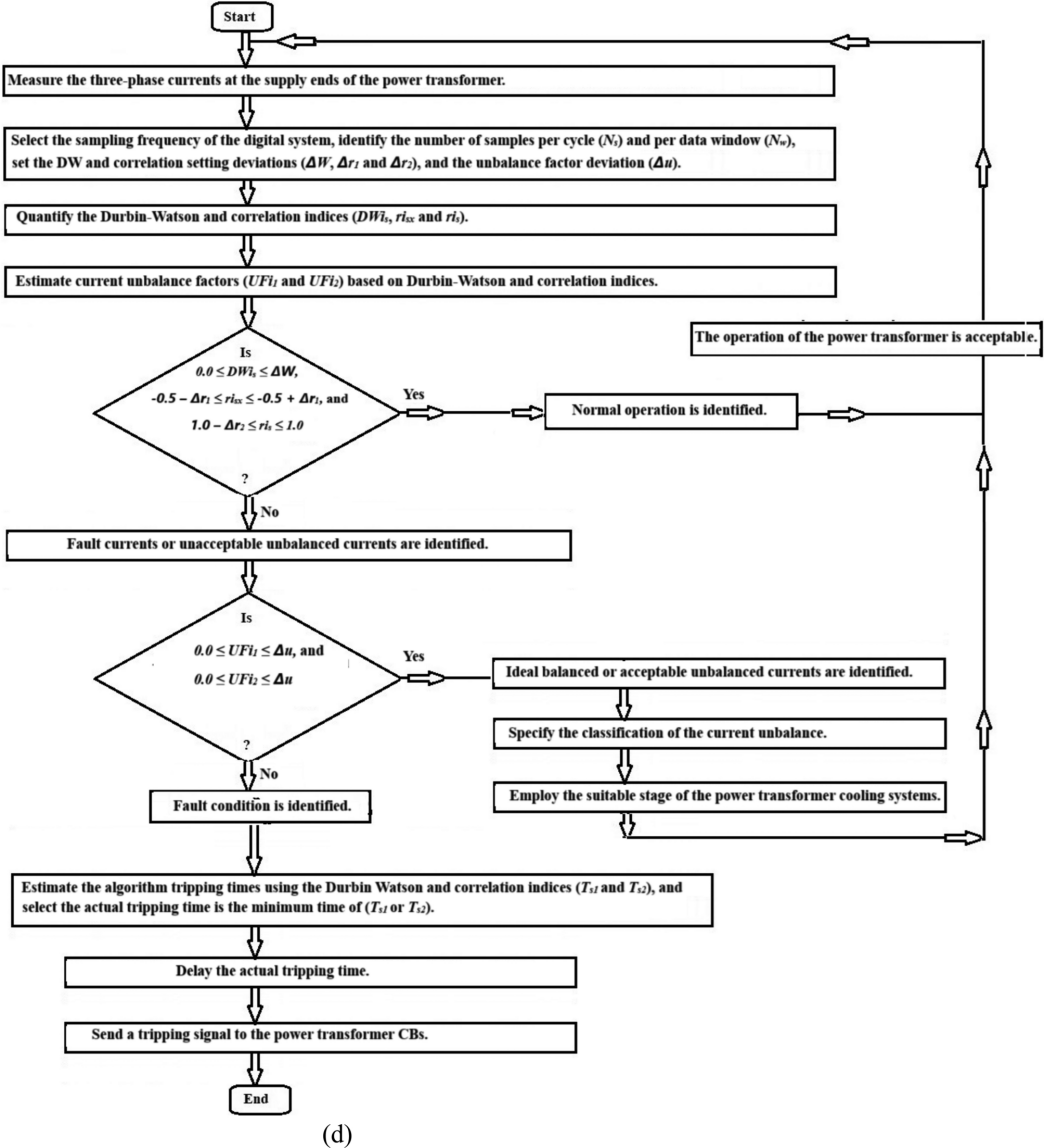
General flow chart of power transformer fault detection and imbalance assessment using the Durbin-Watson and correlation factors.

As illustrated in Figs. 4, 5 and 6, the correlation-based algorithm can be implemented as given below.

1. Take the measurements of the three-phase currents (*i*_*a*_*,, i*_*b*_, and *i*_*c*_) using the current transducers built at the supply ends of the power transformer,
2. Specify the sampling frequency of the digital system, then transform the continuous current signals into numerical values,
3. Determine the quantity of samples per a single cycle (*N*_*s*_) and the quantity of samples per data window (*N*_*w*_),
4. Select the setting values (*Δr*_*1*_ and *Δr*_*2*_) of the cross-correlation and auto-correlation,
5. Compute the three cross-correlation estimators (*ri*_*ab*_*, ri*_*bc*_, and *ri*_*ca*_), and the three auto-correlation estimators (*ri*_*a*_*, ri*_*b*_, and *ri*_*c*_) for the three-phase current waveforms,
6. Obtain the imbalance coefficients (*UFi*_*ab2*_, *UFi*_*bc2*_*, UFi*_*ca2*_, and *UFi*_*2*_) based on the cross-correlation estimators to confirm the event of loss-of-balance, and categorize the intensity of the imbalance into various classifications,
7. Follow the proposed rules based on the correlation factors estimated for three-phase phase currents, as listed in Table 2,
8. Discriminate between healthy and faulty instances for the power transformer*,* as demonstrated in Table 2, when the three auto-correlation coefficients are almost + 1.0, the power transformer situation is healthy. Otherwise, its state is faulty,
9. Differentiate between the states of symmetrical and unsymmetrical currents*,* as illustrated in Table 2, When the three cross-correlation coefficients are nearly − 0.5, the transformer currents are symmetrical. Otherwise, unsymmetrical currents are identified,
10. Quantify the relay tripping time (*T*_*t2*_) based on the auto-correlation coefficients calculated for three-phase currents,
11. Issue a tripping flag to the annunciator board, and send an isolation permission to the transformer circuit breakers to avoid its damage. This procedure is carried out when the transformer is susceptible to an inter-turn, earth or phase fault,
12. The data set size (*N*_*w*_) and the correlation pre-setting deviations (*Δr*_*1*_ and *Δr*_*2*_) can be used to modify the sensitivity, security, and response time of the protection approach,
13. In this algorithm, the correlation pre-setting deviations (*Δr*_*1*_ and *Δr*_*2*_) should be between 0.0 and + 0.20, with a step value of 0.01. The correlation settings can be precisely selected to align with the actual operating conditions of the power system component. The correlation pre-setting deviations (*Δr*_*1*_ and *Δr*_*2*_) are set to + 0.10 and + 0.05, respectively.

#### Protection algorithm sensitivity

Table 3 demonstrates the strategy used to regulate the protection sensitivity of both the Durbin-Watson and correlation algorithms.

**Table 3:** The sensitivity analysis of both the Durbin-Watson and correlation algorithms.

Algorithm designation	Ideal quantity in the case of normal operation	Regulation of algorithm sensitivity	The factors that affect the protection sensitivity	The factors effect on the algorithm tripping time and accuracy
1. Algorithm 1: Durbin-Watson coefficient (*DWi*_*s*_)	0.0	$$Snsv_{1} \begin{array}{*{20}c} {} \\ {} \\ \end{array} \alpha \begin{array}{*{20}c} {} \\ {} \\ \end{array} \frac{{\left| {} \right.DWi_{s} \left. {} \right|}}{{DW_{pu} }}\begin{array}{*{20}c} {} & {} \\ \end{array}$$ The selected *DW*_*pu*_ = + 0.10	$$\uparrow Snsv_{1} \begin{array}{*{20}c} {} \\ {} \\ \end{array} \alpha \begin{array}{*{20}c} {} \\ {} \\ \end{array} \frac{{\left| {} \right.DWi_{s} \left. {} \right| \uparrow }}{{DW_{pu} }}\begin{array}{*{20}c} {} & {} \\ \end{array} or$$ $$\uparrow Snsv_{1} \begin{array}{*{20}c} {} \\ {} \\ \end{array} \alpha \begin{array}{*{20}c} {} \\ {} \\ \end{array} \frac{{\left| {} \right.DWi_{s} \left. {} \right|}}{{DW_{pu} \downarrow }}\begin{array}{*{20}c} {} & {} \\ \end{array}$$	$$\downarrow T_{s1} \begin{array}{*{20}c} {} \\ {} \\ \end{array} \alpha \frac{1}{{ \uparrow Snsv_{1} }}\begin{array}{*{20}c} {} & {} \\ \end{array}$$ $$\downarrow Acc_{1} \begin{array}{*{20}c} {} \\ {} \\ \end{array} \alpha \frac{1}{{ \uparrow Snsv_{1} }}\begin{array}{*{20}c} {} & {} \\ \end{array}$$
2. Algorithm 2: Correlation coefficient (*ri*_*s*_)	1.0	$$Snsv_{2} \begin{array}{*{20}c} {} \\ {} \\ \end{array} \alpha \begin{array}{*{20}c} {} \\ {} \\ \end{array} \frac{{1.0 + ri_{pu} }}{{1.0 + ri_{s} }}\begin{array}{*{20}c} {} & {} \\ \end{array}$$ The selected *ri*_*pu*_ = *1.0 – Δr*_*2*_ = *0.95*	$$\uparrow Snsv_{2} \begin{array}{*{20}c} {} \\ {} \\ \end{array} \alpha \begin{array}{*{20}c} {} \\ {} \\ \end{array} \frac{{1.0 + ri_{pu} \uparrow }}{{1.0 + \begin{array}{*{20}c} {} \\ {} \\ \end{array} ri_{s} }}\begin{array}{*{20}c} {} & {} \\ \end{array} or$$ $$\uparrow Snsv_{2} \begin{array}{*{20}c} {} \\ {} \\ \end{array} \alpha \begin{array}{*{20}c} {} \\ {} \\ \end{array} \frac{{1.0 + ri_{pu} }}{{1.0 + ri_{s} \downarrow }}\begin{array}{*{20}c} {} & {} \\ \end{array}$$	$$\downarrow T_{s2} \begin{array}{*{20}c} {} \\ {} \\ \end{array} \alpha \frac{1}{{ \uparrow Snsv_{2} }}\begin{array}{*{20}c} {} & {} \\ \end{array}$$ $$\downarrow Acc_{2} \begin{array}{*{20}c} {} \\ {} \\ \end{array} \alpha \frac{1}{{ \uparrow Snsv_{2} }}\begin{array}{*{20}c} {} & {} \\ \end{array}$$

The arrow ↑ denotes an increase in the quantity, and the arrow ↓ denotes a decrease in the quantity.

The algorithm sensitivity can be adjusted using the data window size or the settings of the Durbin-Watson (DW) and correlation (*Δr*_*1*_*, Δr*_*2*_*, Δw,* and *Δu*). The protection algorithm will be more sensitive when the setting values (*Δr*_*1*_*, Δr*_*2*_*, Δw,* and *Δu*) are lower. Also, the protection algorithm will become more sensitive when the data window size is lower.

The data window size and the setting deviations of the Durbin-Watson (DW) and correlation (*Δr*_*1*_*, Δr*_*2*_*, Δw,* and *Δu*) have the subsequent effects on the protection characteristics (such as security and sensitivity). As a result, a compromise in the relay settings is still required to ensure coordination between the security and sensitivity of the protection.

#### Protection algorithm speed

The algorithm speed can be readily adjusted by setting the width of the data window used to calculate the Durbin-Watson (DW) and correlation coefficients, which can be specified to be shorter than or equal to a single cycle of the foundation frequency. Thus, the computation time for these coefficients is manageable. The smaller the data window dimension, the faster the algorithm speed, and the lower its accuracy. As a consequence, a balance should be made between the speed and accuracy of the algorithm. High-speed schemes tend to be less accurate, since they have a limited amount of information to make a tripping decisions for electrical equipment.

## Laboratory system under test

**Fig. 7:**
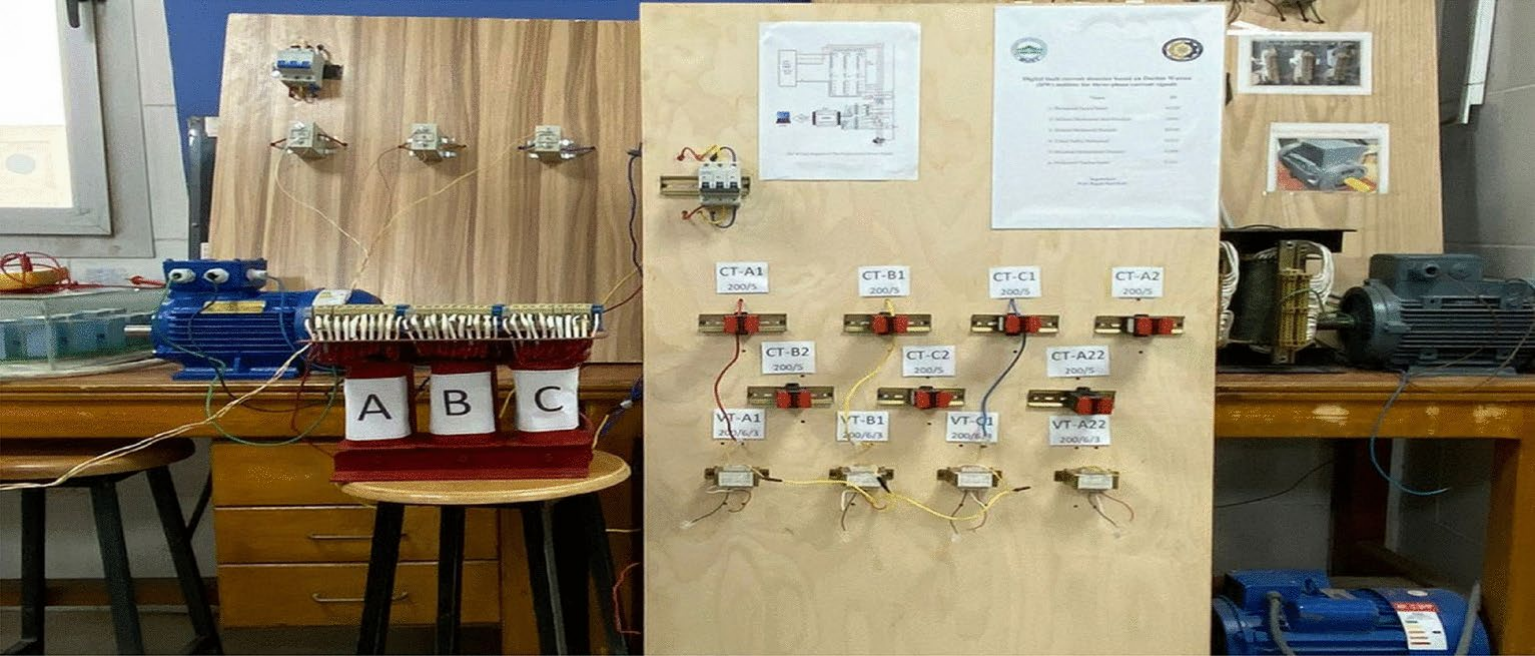
The photograph of the laboratory apparatuses.

**Fig. 8:**
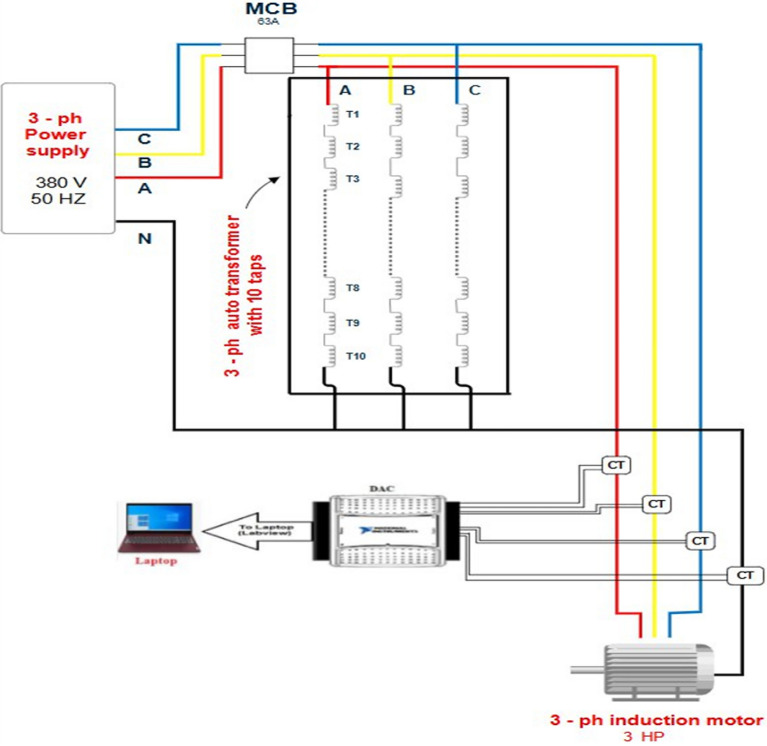
The circuit layout of the laboratory system.

The laboratory power system is established to examine the effectiveness of the suggested scheme based on the dual functions of Durbin-Watson and correlation. This system is composed of a three-phase power supply of 380 V, a three-phase breaker with a rating of 63 A, a three-phase auto-transformer with a rating of 4 kVA, a three-phase asynchronous motor, and three single-phase current transducers with turns’ ratio of 200/5. The current transducers are installed at the supply ends of the primary windings of the power transformer. Each winding of the three-phase power transformer contains 100 turns, including ten taps per phase. This setup enables researchers to conduct comprehensive experiments under diverse types of faults and operating conditions to make sure the validity of the protection plan. Faults related to turn-to-turn, phase-to-phase and phase-to-neutral will be implemented in this study. The three single-phase current transducers are used to transform the currents from the auto-transformer primary windings into secondary currents that serve as the analog input to an analog-to-digital converter called DAC. A hardware part of the digital protective relay is comprised of a personal computer and the DAC^40,41^. The USB-6009 is the DAC from the National Instruments, which converts continuous current waveforms into discrete values, is set to function in differential mode. The protection algorithms are processed using the LABVIEW software package, which acquires the discrete values from the DAC. Figure 7 depicts the photograph of the laboratory apparatuses. The circuit layout of the experimental system is illustrated in Fig. 8. The parameter specifications of the system apparatuses are provided in Appendix 1. The numerical values used in the protection system are listed in Appendix 2.

## Experiments and statistical analysis

In this section, the quantitative results from different fault events will be analyzed and interpreted. For each experimental investigation, the estimated Durbin-Watson and correlation factors for the three-phase currents of the primary windings of the power transformer will be discussed in more detail.

### Experiment 1: winding-to-neutral fault (C1-N)

**Fig. 9:**
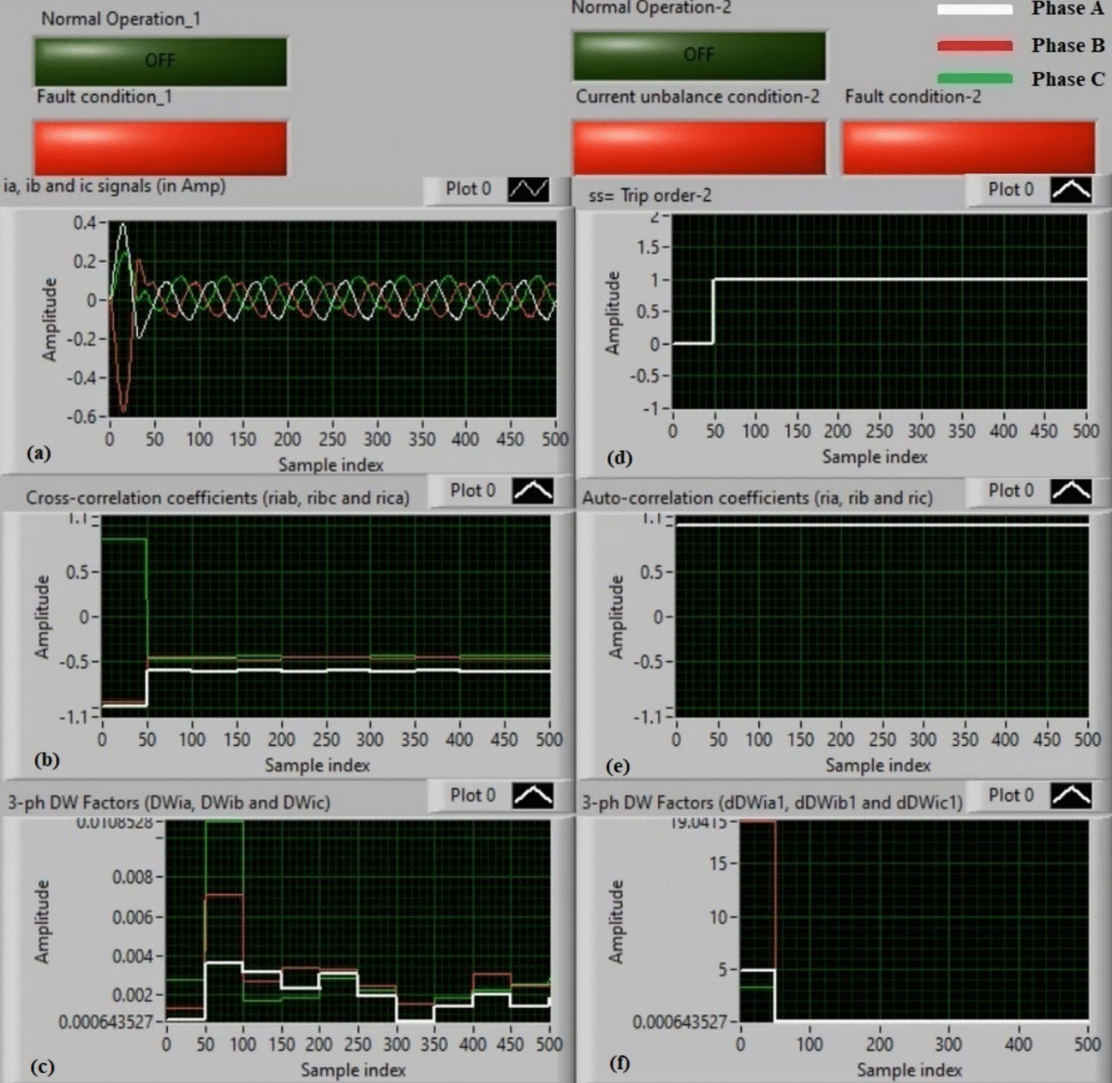
Algorithm results for experiment 1. (**a**) Three-phase currents (*i*_*a*_*(k), i*_*b*_*(k),* and *i*_*c*_*(k)*), (**b**) Three cross-correlation coefficients (*ri*_*ab*_*, ri*_*bc*_*,* and *ri*_*ca*_), (**c**) Durbin-Watson factors (*DWi*_*a*_*, DWi*_*b*_*,* and *DWi*_*c*_), (**d**) Tripping signal, (**e**) Three auto-correlation coefficients (*ri*_*a*_*, ri*_*b*_*,* and *ri*_*c*_), and (**f**) Durbin-Watson factors (*dDWi*_*a1*_*, dDWi*_*b1*_*,* and *dDWi*_*c1*_).

Figures 9 show algorithm results for experiment 1. This case is a phase-to-neutral fault (*C1-N*). Figure 9a introduces three-phase currents (*i*_*a*_*, i*_*b*_, and *i*_*c*_) measured at the load side of the power transformer, and Fig. 9b presents the three cross-correlation factors (*ri*_*ab*_*, ri*_*bc*_*,* and *ri*_*ca*_) computed for the three currents. Figure 9c illustrates the Durbin-Watson factors (*DWi*_*a*_*, DWi*_*b*_*,* and *DWi*_*c*_) calculated for the three currents. Figure 9d shows a tripping signal, which is + 1.0 after the fault occurrence of the display time, and Fig. 9e illustrates the three auto-correlation factors (*ri*_*a*_*, ri*_*b*_*,* and *ri*_*c*_) quantified for the three currents. Figure 9f depicts the Durbin-Watson factors (*dDWi*_*a1*_*, dDWi*_*b1*_*,* and *dDWi*_*c1*_) estimated for the three currents.

The algorithm results affirm that the power transformer state is faulty, as shown in Fig. 9. The values of the Durbin-Watson factors are considerably larger than *ΔW* =  + 0.1 during the first cycle of the display time. Also, the operating points of the three cross-correlation coefficients are situated within the tripping zones. Therefore, the Durbin-Watson and correlation-driven current relay will be activated when the winding-to-neutral fault (*C1-N*) occurs. As a result, the protection scheme responds rapidly. It operates after about 5.25 ms.

### Experiment 2: winding-to-neutral fault (C2-N)

**Fig. 10:**
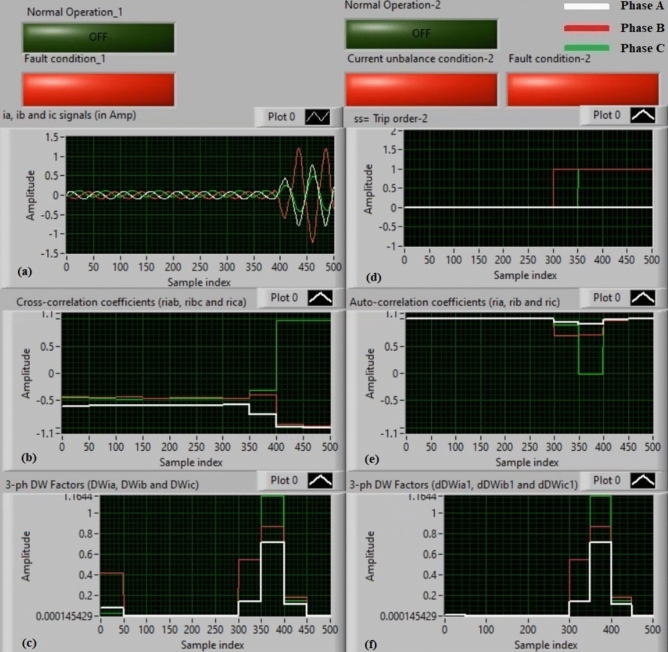
Algorithm results for experiment 2. (**a**) Three-phase currents (*i*_*a*_*(k), i*_*b*_*(k),* and *i*_*c*_*(k)*), (**b**) Three cross-correlation coefficients (*ri*_*ab*_*, ri*_*bc*_*,* and *ri*_*ca*_), (**c**) Durbin-Watson factors (*DWi*_*a*_*, DWi*_*b*_*,* and *DWi*_*c*_), (**d**) Tripping signal, (**e**) Three auto-correlation coefficients (*ri*_*a*_*, ri*_*b*_*,* and *ri*_*c*_), and (**f**) Durbin-Watson factors (*dDWi*_*a1*_*, dDWi*_*b1*_*,* and *dDWi*_*c1*_).

Figure 10 illustrate testing results in the instance of a phase-to-neutral fault (*C2-N*). Figure 10a offers three-phase currents (*i*_*a*_*, i*_*b*_, and *i*_*c*_) taken at the load end of the power transformer, and Fig. 10b exhibits the three cross-correlation estimators (*ri*_*ab*_*, ri*_*bc*_*,* and *ri*_*ca*_) calculated for the three currents. Figure 10c demonstrates the Durbin-Watson factors (*DWi*_*a*_*, DWi*_*b*_*,* and *DWi*_*c*_) computed for the three currents. Figure 10d depicts a tripping signal, whose value is + 1.0 during the fault presence of the display period. Figure 10e presents the three auto-correlation estimators (*ri*_*a*_*, ri*_*b*_*,* and *ri*_*c*_) estimated for the three currents. Figure 10f shows the Durbin-Watson factors (*dDWi*_*a1*_*, dDWi*_*b1*_*,* and *dDWi*_*c1*_) quantified for the three currents.

The recorded results ensure that the fault is located on the auto-transformer, as shown in Fig. 10. The quantities of the Durbin-Watson factors increase significantly, where they are greater than the threshold value (*ΔW* =  + 0.1) during the fault span. Moreover, the operating points of both cross-correlation and auto-correlation coefficients are existent in the tripping regions. Thus, the Durbin-Watson and correlation-based current relay responds quickly to the situation of the winding-to-neutral fault (*C2-N*). As a consequence, the operating time of the protection scheme is roughly 85.91 ms.

### Experiment 3: winding-to-neutral fault (C3-N))

**Fig. 11:**
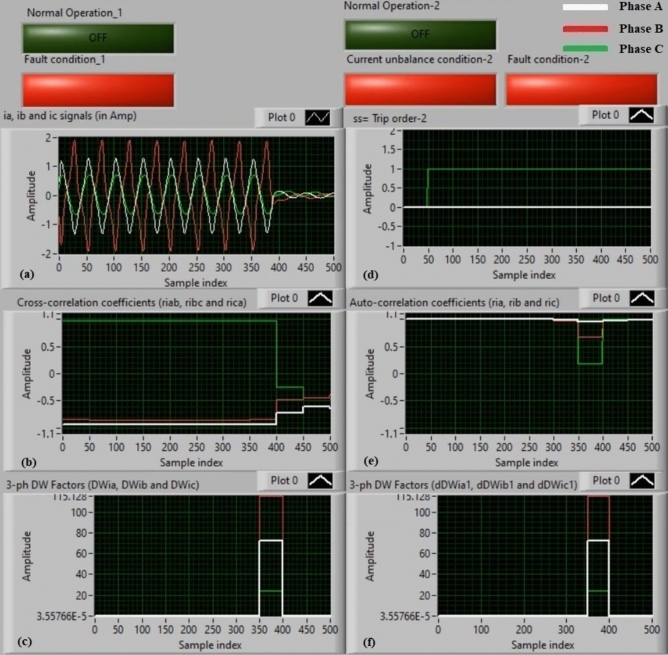
Algorithm results for experiment 3. (**a**) Three-phase currents (*i*_*a*_*(k), i*_*b*_*(k),* and *i*_*c*_*(k)*), (**b**) Three cross-correlation coefficients (*ri*_*ab*_*, ri*_*bc*_*,* and *ri*_*ca*_), (**c**) Durbin-Watson factors (*DWi*_*a*_*, DWi*_*b*_*,* and *DWi*_*c*_), (**d**) Tripping signal, (**e**) Three auto-correlation coefficients (*ri*_*a*_*, ri*_*b*_*,* and *ri*_*c*_), and (**f**) Durbin-Watson factors (*dDWi*_*a1*_*, dDWi*_*b1*_*,* and *dDWi*_*c1*_).

Figure 11 introduce protection results for experiment 3. This case is a phase-to-neutral fault (*C3-N*). Figure 11a depicts three-phase currents (*i*_*a*_*, i*_*b*_*,* and *i*_*c*_) for the load terminals of the auto-transformer, and Fig. 11b offers the three cross-correlation coefficients (*ri*_*ab*_*, ri*_*bc*_*,* and *ri*_*ca*_) quantified for the three currents. Figure 11c exhibits the Durbin-Watson factors (*DWi*_*a*_*, DWi*_*b*_*,* and *DWi*_*c*_) estimated for the three currents. Figure 11d depicts a tripping signal, whose value equals to + 1.0 during the fault time of the display period. Figure 11e describes the three auto-correlation coefficients (*ri*_*a*_*, ri*_*b*_*,* and *ri*_*c*_) computed for the three currents. Figure 11f presents the Durbin-Watson factors (*dDWi*_*a1*_*, dDWi*_*b1*_*,* and *dDWi*_*c1*_) calculated for the three currents.

The recorded outcomes indicate that the short-circuit incidence is existent on the power transformer, as illustrated in Fig. 11. The quantities of the Durbin-Watson factors exceed significantly, where they are greater than the setting value (*ΔW* =  + 0.1) during the fault time period. Moreover, the operating points of both cross-correlation and auto-correlation estimators are within the tripping areas. Consequently, the current relay based on the Durbin-Watson and correlation functions is active in the event of the winding-to-neutral fault (*C3-N*). Hence, the suggested scheme operates quickly. The protection scheme takes nearly 0.868 ms to send the tripping flag. In this case, it is obvious that the tripping time is instantaneous because the Durbin-Watson factors record extremely large values. The Durbin-Watson and correlation-based protection scheme is beneficial to detect faults, and estimate the appropriate tripping time for isolating the fault occurrence.

### Experiment 4: winding-to-neutral fault (B1-N)

**Fig. 12:**
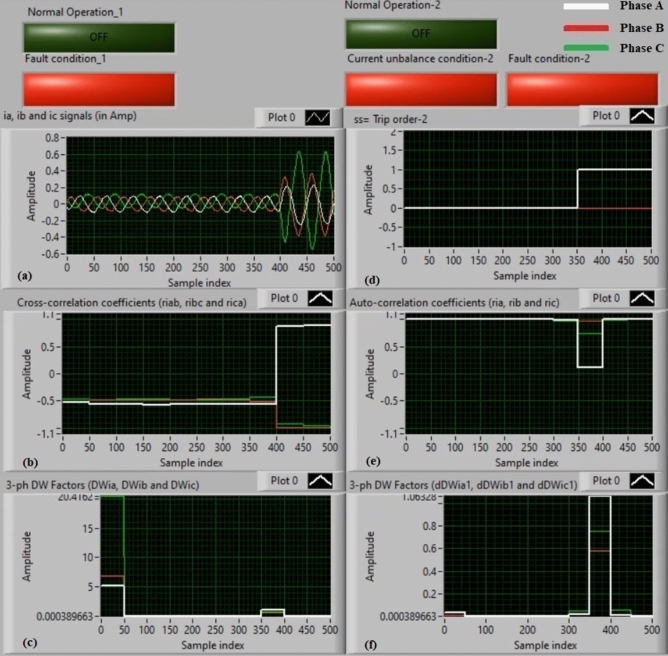
Algorithm results for experiment 4. (**a**) Three-phase currents (*i*_*a*_*(k), i*_*b*_*(k),* and *i*_*c*_*(k)*), (**b**) Three cross-correlation coefficients (*ri*_*ab*_*, ri*_*bc*_*,* and *ri*_*ca*_), (**c**) Durbin-Watson factors (*DWi*_*a*_*, DWi*_*b*_*,* and *DWi*_*c*_), (**d**) Tripping signal, (**e**) Three auto-correlation coefficients (*ri*_*a*_*, ri*_*b*_*,* and *ri*_*c*_), and (**f**) Durbin-Watson factors (*dDWi*_*a1*_*, dDWi*_*b1*_*,* and *dDWi*_*c1*_).

Figure 12 show the experimental results of case study 4. This experiment is a phase-to-neutral fault (*B1-N*). Figure 12a manifests three-phase currents (*i*_*a*_*, i*_*b*_*,* and *i*_*c*_) for the secondary side of the power transformer, and Fig. 12b exhibits the three cross-correlation coefficients (*ri*_*ab*_*, ri*_*bc*_*,* and *ri*_*ca*_) quantified for the three currents. Figure 12c exhibits the Durbin-Watson factors (*DWi*_*a*_*, DWi*_*b*_*,* and *DWi*_*c*_) evaluated for the three currents. Figure 12d illustrates a tripping signal, which indicates + 1.0 during the fault interval of the display time. Figure 12e describes the three auto-correlation coefficients (*ri*_*a*_*, ri*_*b*_*,* and *ri*_*c*_) computed for the three currents. Figure 12f presents the Durbin-Watson factors (*dDWi*_*a1*_*, dDWi*_*b1*_*,* and *dDWi*_*c1*_) calculated for the three currents.

The experimental outcomes affirm that the short-circuit current is present on the power transformer, as described in Fig. 12. The quantities of the Durbin-Watson factors are much stronger than the pickup value (*ΔW* =  + 0.1) during the fault time distance. Furthermore, the operating points of both cross-correlation and auto-correlation coefficients are inside the tripping regions. As a result, the current relay based on the Durbin-Watson and correlation algorithms is feasible in the incidence of the winding-to-neutral fault (*B1-N*). Therefore, the suggested scheme works quickly. The estimated time of the relay operation is approximately 94.07 ms.

### Experiment 5: winding-to-neutral fault (B2-N)

**Fig. 13:**
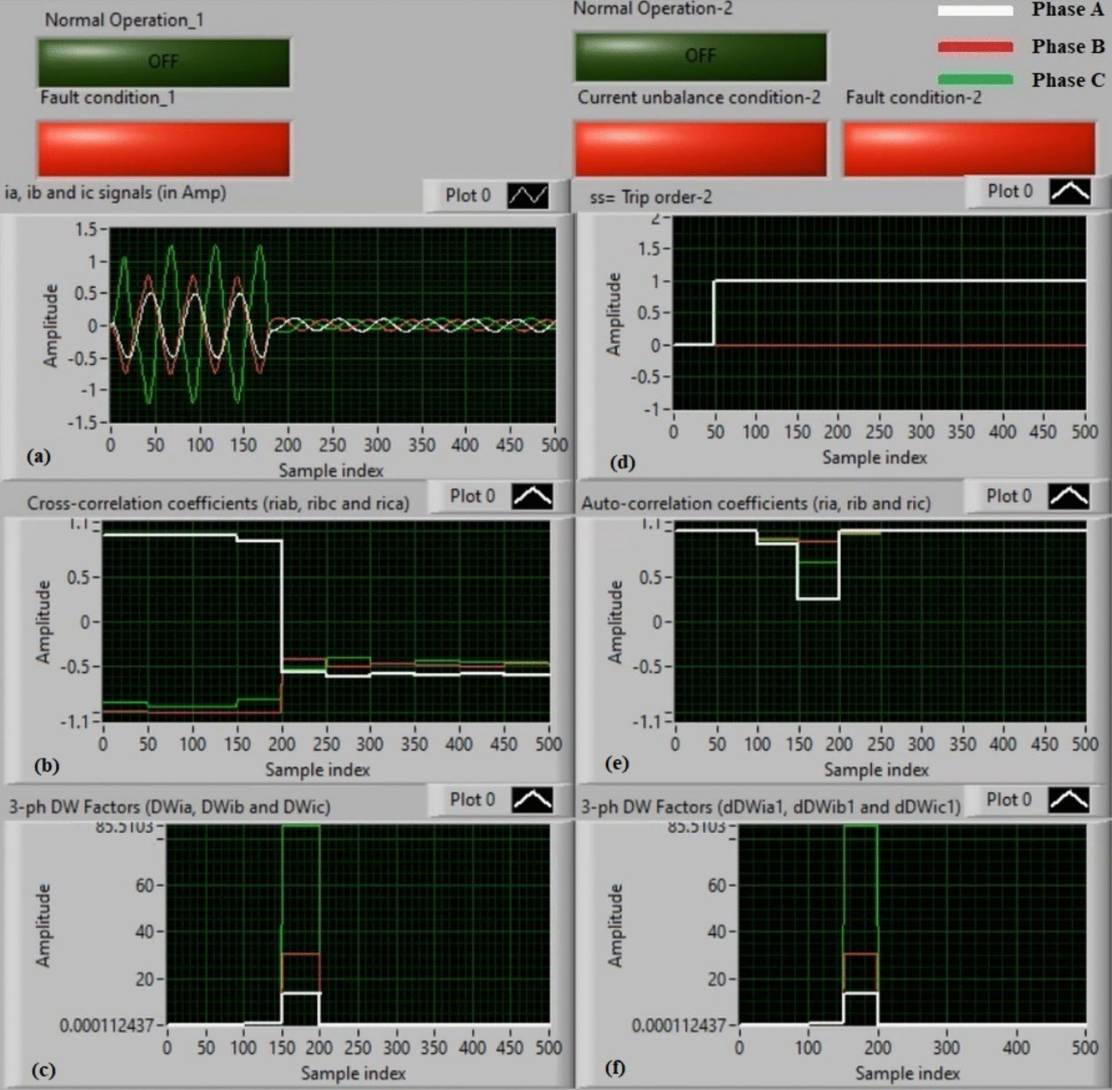
Algorithm results for experiment 5. (**a**) Three-phase currents (*i*_*a*_*(k), i*_*b*_*(k),* and *i*_*c*_*(k)*), (**b**) Three cross-correlation coefficients (*ri*_*ab*_*, ri*_*bc*_*,* and *ri*_*ca*_), (**c**) Durbin-Watson factors (*DWi*_*a*_*, DWi*_*b*_*,* and *DWi*_*c*_), (**d**) Tripping signal, (**e**) Three auto-correlation coefficients (*ri*_*a*_*, ri*_*b*_*,* and *ri*_*c*_), and (**f**) Durbin-Watson factors (*dDWi*_*a1*_*, dDWi*_*b1*_*,* and *dDWi*_*c1*_).

Figure 13 manifest the experimental results of test 5. This experiment is a phase-to-neutral fault (*B2-N*). Figure 13a exhibits three-phase secondary currents (*i*_*a*_*, i*_*b*_*,* and *i*_*c*_). Figure 13b illustrates three cross-correlation coefficients (*ri*_*ab*_*, ri*_*bc*_*,* and *ri*_*ca*_), and Fig. 13c shows Durbin-Watson factors (*DWi*_*a*_*, DWi*_*b*_*,* and *DWi*_*c*_). Figure 13d plots a tripping signal, which denotes the value of + 1.0 during the fault interval. Figure 13e illustrates three auto-correlation coefficients (*ri*_*a*_*, ri*_*b*_*,* and *ri*_*c*_), and Fig. 13f demonstrates the Durbin-Watson factors (*dDWi*_*a1*_*, dDWi*_*b1*_*,* and *dDWi*_*c1*_).

The testing results prove that the short-circuit event is situated on the power transformer windings, as shown in Fig. 13. The values of the Durbin-Watson factors increase suddenly, and they are larger than the pickup value (*ΔW* =  + 0.1) during the fault time distance. The operating points of both cross-correlation and auto-correlation factors are existent within the tripping areas. Consequently, the current protection based on the Durbin-Watson and correlation statistics is feasible in the event of the winding-to-neutral fault (*B2-N*). Therefore, the protection algorithms are highly swift. The appropriate time of the relay operation is approximately 1.170 ms. In this particular instance, it is clear that the tripping time is instantaneous because the Durbin-Watson factors change to exceptionally high values.

### Experiment 6: turn-to-turn fault (A1-A2)

**Fig. 14:**
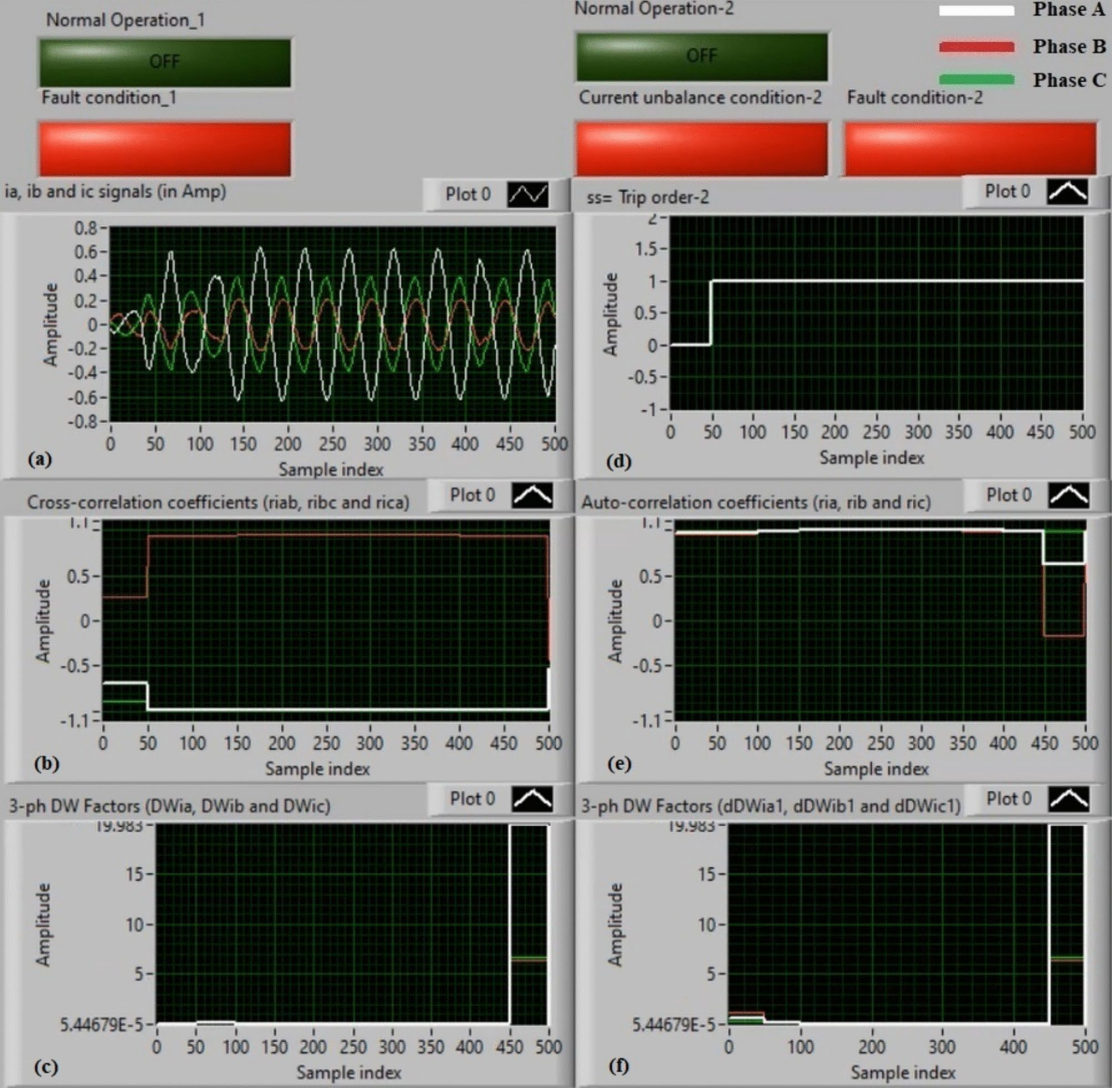
Algorithm results for experiment 6. (**a**) Three-phase currents (*i*_*a*_*(k), i*_*b*_*(k),* and *i*_*c*_*(k)*), (**b**) Three cross-correlation coefficients (*ri*_*ab*_*, ri*_*bc*_*,* and *ri*_*ca*_), (**c**) Durbin-Watson factors (*DWi*_*a*_*, DWi*_*b*_*,* and *DWi*_*c*_), (**d**) Tripping signal, (**e**) Three auto-correlation coefficients (*ri*_*a*_*, ri*_*b*_*,* and *ri*_*c*_), and (**f**) Durbin-Watson factors (*dDWi*_*a1*_*, dDWi*_*b1*_*,* and *dDWi*_*c1*_).

Figure 14 exhibit the results obtained from experiment 6. This test is a turn-to-turn fault (*A1–A2*). The three-phase secondary currents (*i*_*a*_*, i*_*b*_*,* and *i*_*c*_) are illustrated in Fig. 14a. The three cross-correlation estimators (*ri*_*ab*_*, ri*_*bc*_*,* and *ri*_*ca*_) are depicted in Fig. 14b, and the three Durbin-Watson factors (*DWi*_*a*_*, DWi*_*b*_*,* and *DWi*_*c*_) are shown in Fig. 14c. Figure 14d presents the tripping signal, which signifies the value of + 1.0 during the fault period of the full display time. Figure 14e manifests the three auto-correlation estimators (*ri*_*a*_*, ri*_*b*_*,* and *ri*_*c*_), and the three Durbin-Watson factors (*dDWi*_*a1*_*, dDWi*_*b1*_*,* and *dDWi*_*c1*_) are presented in Fig. 14f.

The testing outcomes demonstrate that the short-circuit current originates on the power transformer windings, as described in Fig. 14. The values of the Durbin-Watson factors raise suddenly, and they are higher than the pickup value (*ΔW* =  + 0.1) during the fault time distance. The operating points of all cross-correlation and auto-correlation coefficients are located within the tripping zones. The proposed relay based on the Durbin-Watson and correlation is active in the incidence of the turn-to-turn fault (*A1–A2*). Consequently, the protection algorithm responds speedily. In this incidence, the protection scheme should start working after the fault presence of 5.0 ms.

### Experiment 7: turn-to-turn fault (A2–A3)

**Fig. 15:**
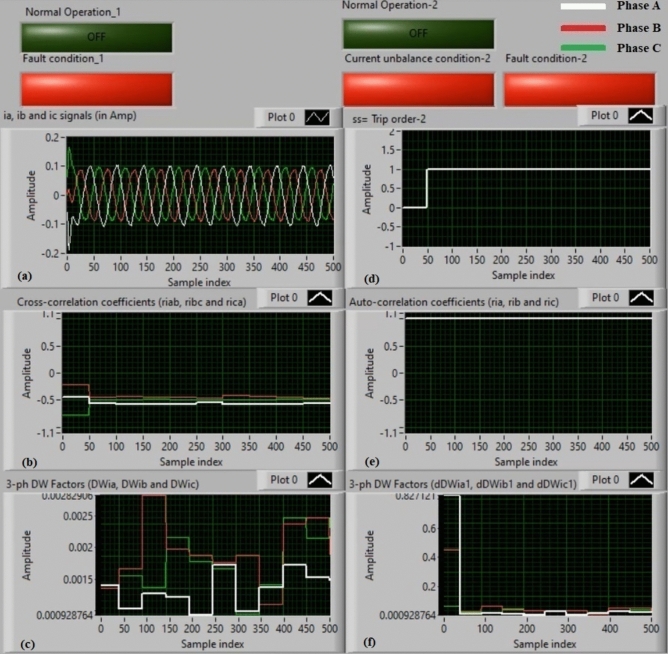
Algorithm results for experiment 7. (**a**) Three-phase currents (*i*_*a*_*(k), i*_*b*_*(k),* and *i*_*c*_*(k)*), (**b**) Three cross-correlation coefficients (*ri*_*ab*_*, ri*_*bc*_*,* and *ri*_*ca*_), (**c**) Durbin-Watson factors (*DWi*_*a*_*, DWi*_*b*_*,* and *DWi*_*c*_), (**d**) Tripping signal, (**e**) Three auto-correlation coefficients (*ri*_*a*_*, ri*_*b*_*,* and *ri*_*c*_), and (**f**) Durbin-Watson factors (*dDWi*_*a1*_*, dDWi*_*b1*_*,* and *dDWi*_*c1*_).

Figure 15 introduce the results recorded for experiment 7. This test is a turn-to-turn fault (*A2–A3*). The three-phase secondary currents (*i*_*a*_*, i*_*b*_*,* and *i*_*c*_) are depicted in Fig. 15a. The three cross-correlation estimators (*ri*_*ab*_*, ri*_*bc*_*,* and *ri*_*ca*_) are illustrated in Fig. 15b, and the three Durbin-Watson factors (*DWi*_*a*_*, DWi*_*b*_*,* and *DWi*_*c*_) are shown in Fig. 15c. Figure 15d presents the tripping signal, which signifies that its value is + 1.0 during the fault period of the display time. Figure 15e manifests the three auto-correlation estimators (*ri*_*a*_*, ri*_*b*_*,* and *ri*_*c*_), and the three Durbin-Watson factors (*dDWi*_*a1*_*, dDWi*_*b1*_*,* and *dDWi*_*c1*_) are demonstrated in Fig. 15f.

The produced results manifest that the short-circuit current is generated within the power transformer windings, as shown in Fig. 15. The quantities of the Durbin-Watson factors intensify suddenly, and they are larger than the setting value (*ΔW* =  + 0.1) during the fault presence. The operating points of all cross-correlation and auto-correlation quantities are within the tripping regions. Therefore, the developed relay based on the Durbin-Watson and correlation estimators is dynamic in the situation of the turn-to-turn fault (*A2–A3*). Consequently, the protection algorithm reacts quickly. In this case, the protective relay should send a tripping alarm after a time period of 120.92 ms following the fault inception.

### Experiment 8: winding-to-winding fault (B1-C1)

**Fig. 16:**
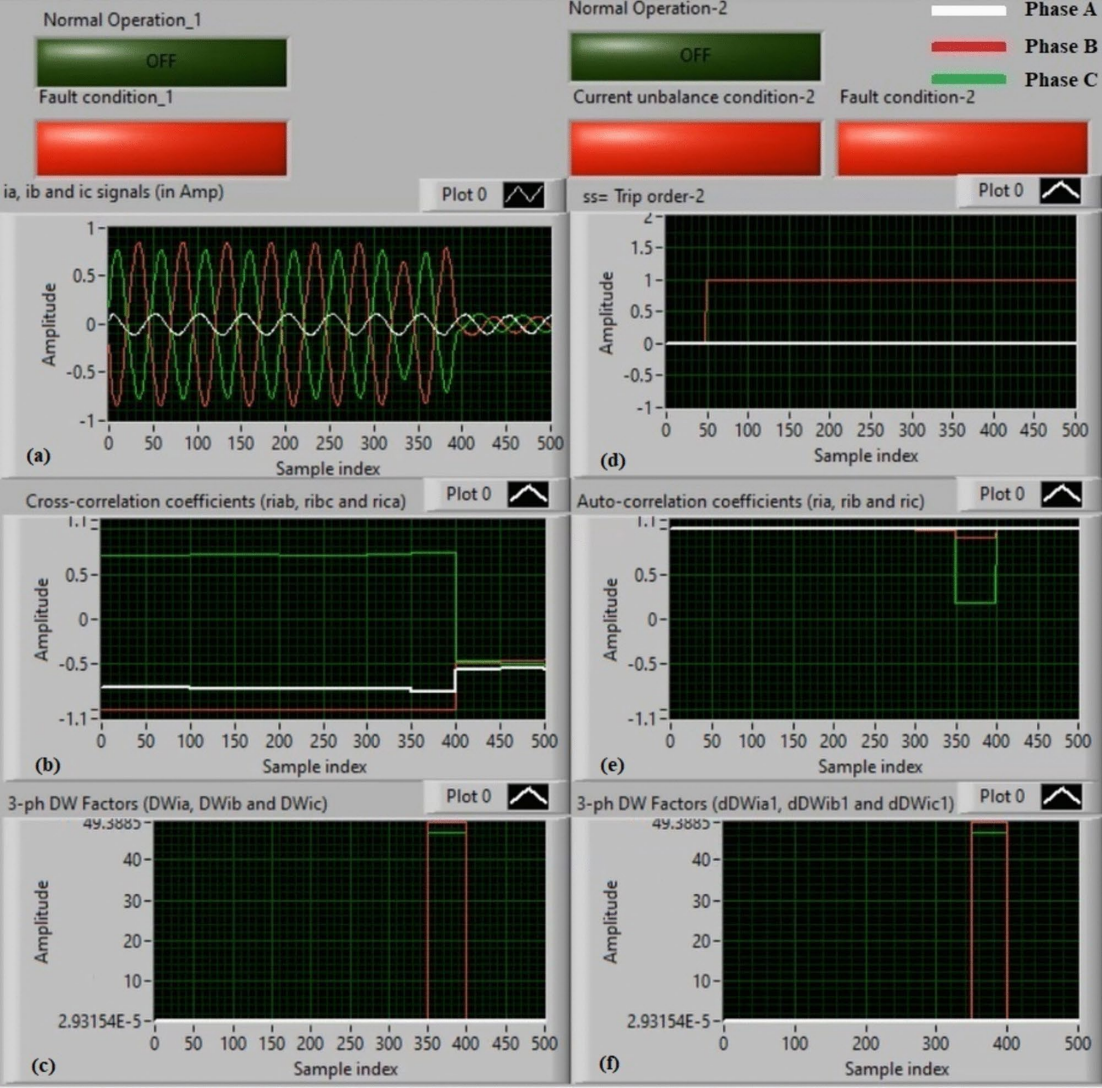
Algorithm results for experiment 8. (**a**) Three-phase currents (*i*_*a*_*(k), i*_*b*_*(k),* and *i*_*c*_*(k)*), (**b**) Three cross-correlation coefficients (*ri*_*ab*_*, ri*_*bc*_*,* and *ri*_*ca*_), (**c**) Durbin-Watson factors (*DWi*_*a*_*, DWi*_*b*_*,* and *DWi*_*c*_), (**d**) Tripping signal, (**e**) Three auto-correlation coefficients (*ri*_*a*_*, ri*_*b*_*,* and *ri*_*c*_), and (**f**) Durbin-Watson factors (*dDWi*_*a1*_*, dDWi*_*b1*_*,* and *dDWi*_*c1*_).

Figure 16 describe the practical results of test 8. This experiment is a phase-to-phase fault (*B1–C1*). Figure 16a demonstrates the three-phase secondary currents (*i*_*a*_*, i*_*b*_*,* and *i*_*c*_). Figure 16b exhibits the three cross-correlation estimators (*ri*_*ab*_*, ri*_*bc*_*,* and *ri*_*ca*_), and Fig. 16c illustrates the Durbin-Watson factors (*DWi*_*a*_*, DWi*_*b*_*,* and *DWi*_*c*_). The tripping signal is shown in Fig. 16d, and its value is + 1.0 during the fault extent of the full display time. Figure 16e depicts the three auto-correlation estimators (*ri*_*a*_*, ri*_*b*_*,* and *ri*_*c*_), and Fig. 16f manifests the three Durbin-Watson factors (*dDWi*_*a1*_*, dDWi*_*b1*_*,* and *dDWi*_*c1*_).

The results indicate that the fault classification is phase-to-phase (*B1–C1*) for the power transformer windings, as depicted in Fig. 16. This is because *ri*_*bc*_ = − 1.0 during the fault extent, as shown in Fig. 16b. It is seen that the two currents (*i*_*b*_ and *i*_*c*_) of the two faulty phases have sinusoidal shapes with reverse polarity. The two waveforms are free from CT saturation (i.e., not distorted), equal in magnitude and opposite in polarity. The values of the Durbin-Watson factors rise suddenly, surpassing the threshold value (*ΔW* =  + 0.1) during the fault extent. The operating points of the cross-correlation and auto-correlation coefficients are concentrated in the tripping zones. As a result, the protective relay based on the Durbin-Watson and correlation is efficacious in the case of the winding-to-winding fault (*B1–C1*). As a consequence, the suggested protection based on the Durbin-Watson and correlation functions complies rapidly in the event of winding-to-winding shunt fault (*B1–C1*). In this experiment, the algorithm tripping time is almost 2.0 ms.

### Experiment 9: winding-to-winding fault (B2-C2)

**Fig. 17:**
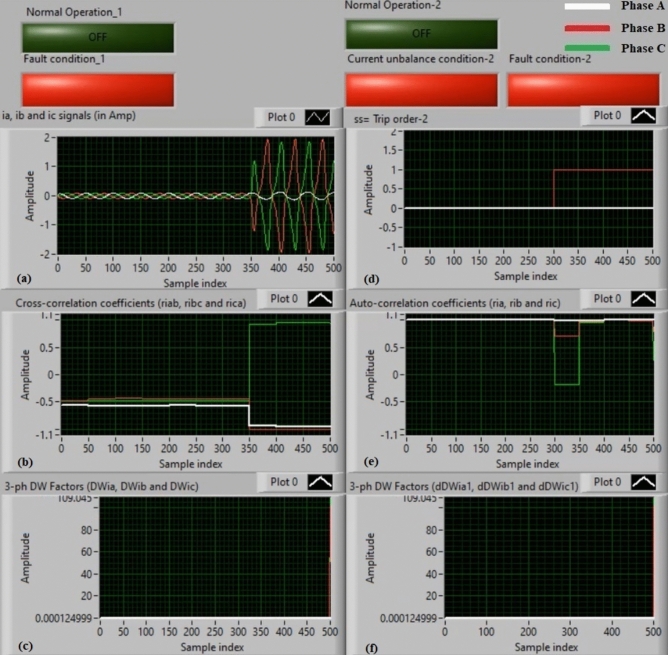
Algorithm results for experiment 9. (**a**) Three-phase currents (*i*_*a*_*(k), i*_*b*_*(k),* and *i*_*c*_*(k)*), (**b**) Three cross-correlation coefficients (*ri*_*ab*_*, ri*_*bc*_*,* and *ri*_*ca*_), (**c**) Durbin-Watson factors (*DWi*_*a*_*, DWi*_*b*_*,* and *DWi*_*c*_), (**d**) Tripping signal, (**e**) Three auto-correlation coefficients (*ri*_*a*_*, ri*_*b*_*,* and *ri*_*c*_), and (**f**) Durbin-Watson factors (*dDWi*_*a1*_*, dDWi*_*b1*_*,* and *dDWi*_*c1*_).

Figure 17 describe the practical results of test 9. This experiment is a winding-to- winding fault (*B2–C2*). Figure 17a introduces three-phase secondary currents (*i*_*a*_*, i*_*b*_*,* and *i*_*c*_). Figure 17b illustrates the three cross-correlation estimators (*ri*_*ab*_*, ri*_*bc*_*,* and *ri*_*ca*_), and Fig. 17c exhibits the Durbin-Watson factors (*DWi*_*a*_*, DWi*_*b*_*,* and *DWi*_*c*_). Figure 17d manifests the tripping signal during the fault extent of the full display time, where its value is + 1.0. Figure 17e illustrates the three auto-correlation estimators (*ri*_*a*_*, ri*_*b*_*,* and *ri*_*c*_), and Fig. 17f presents the Durbin-Watson factors (*dDWi*_*a1*_*, dDWi*_*b1*_*,* and *dDWi*_*c1*_).

The results confirm that the fault description is line-to-line (*B2–C2*) for the power transformer, as depicted in Fig. 17. This is because *ri*_*bc*_ = − 1.0 during the fault presence, as shown in Fig. 17b. It is clear that the two currents (*i*_*b*_ and *i*_*c*_) of two faulty phases have distorted waveforms, arising from a CT saturation conditions of the two faulty phases (*B* and *C*), and they are equal in magnitude and reverse in polarity. The quantities of the Durbin-Watson factors rise suddenly, exceeding the pickup value (*ΔW* =  + 0.1) during the fault time interval. The operating points of both cross-correlation and auto-correlation coefficients are existent within the tripping areas. As a consequence, the suggested relay based on the Durbin-Watson and correlation is effective in the event of the winding-to-winding fault (*B2–C2*). As a result, the suggested protection based on the Durbin-Watson and correlation functions is dynamic quickly in the event of winding-to-winding shunt fault (*B2–C2*). In this experiment, the relay tripping time is about 1.0 ms.

### Experiment 10: Winding-to-winding fault (B3–C3)

**Fig. 18:**
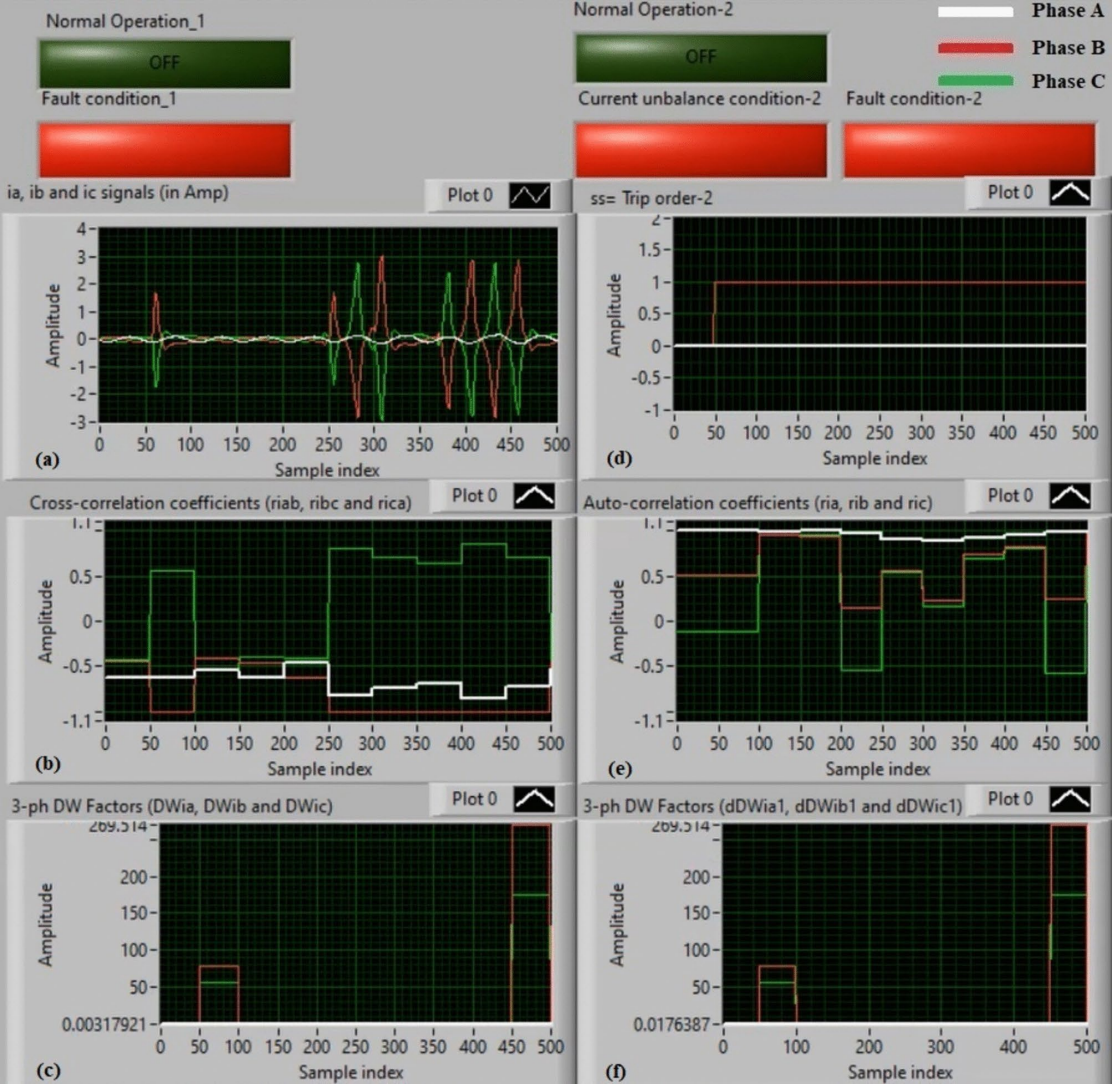
Algorithm results for experiment 10. (**a**) Three-phase currents (*i*_*a*_*(k), i*_*b*_*(k),* and *i*_*c*_*(k)*), (**b**) Three cross-correlation coefficients (*ri*_*ab*_*, ri*_*bc*_*,* and *ri*_*ca*_), (**c**) Durbin-Watson factors (*DWi*_*a*_*, DWi*_*b*_*,* and *DWi*_*c*_), (**d**) Tripping signal, (**e**) Three auto-correlation coefficients (*ri*_*a*_*, ri*_*b*_*,* and *ri*_*c*_), and (**f**) Durbin-Watson factors (*dDWi*_*a1*_*, dDWi*_*b1*_*,* and *dDWi*_*c1*_).

Figure 18 exhibit the experimental results of test 10. This experiment is a coil-to-coil fault (*B3-C3*). Figure 18a illustrates three-phase secondary currents (*i*_*a*_*, i*_*b*_*,* and *i*_*c*_). Figure 18b manifests three cross-correlation estimators (*ri*_*ab*_*, ri*_*bc*_*,* and *ri*_*ca*_), and Fig. 18c describes Durbin-Watson factors (*DWi*_*a*_*, DWi*_*b*_*,* and *DWi*_*c*_). Figure 18d presents the tripping signal during the winding-to-winding fault time, which is equal to one. Figure 18e introduces the three auto-correlation estimators (*ri*_*a*_*, ri*_*b*_*,* and *ri*_*c*_), and Fig. 18f describes the Durbin-Watson factors (*dDWi*_*a1*_*, dDWi*_*b1*_*,* and *dDWi*_*c1*_).

The results prove that the fault form is phase-to-phase (*B3–C3*) for the power transformer windings, as presented in Fig. 18. The values of *ri*_*bc*_ are -1.0 during the fault interval, as shown in Fig. 18b. It is evident that the two faulty currents (*i*_*b*_ and *i*_*c*_) have more distortion in their waveforms due to the extreme CT saturation conditions of the two faulty phases, and they are equal in magnitude and reverse in polarity. The Durbin-Watson factors grow suddenly, overtaking the pickup value (*ΔW* =  + 0.1) during the fault time span. In addition, the operating points of the cross-correlation and auto-correlation factors are existing within the tripping zones. As a consequence, the present relay based on the Durbin-Watson and correlation functions is functional in the event of the winding-to-winding fault (*B3–C3*). As a result, the suggested protection is efficacious rapidly in the event of winding-to-winding fault (*B3–C3*). In this case, the operating time of the protection algorithm is roughly 0.5 ms.

### Experiment 11: Phase ‘A’ loss

**Fig. 19:**
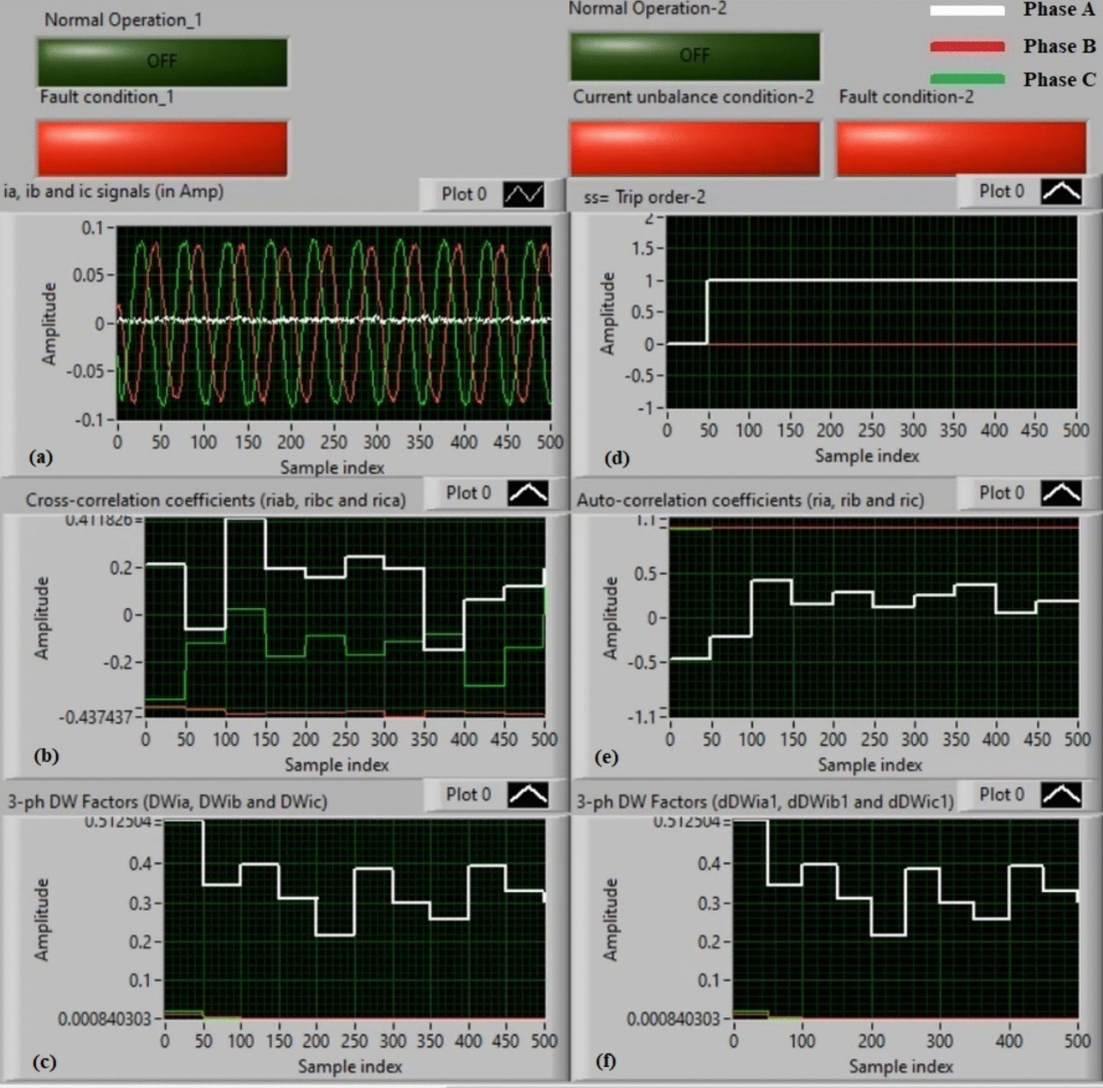
Algorithm results for experiment 11. (**a**) Three-phase currents (*i*_*a*_*(k), i*_*b*_*(k),* and *i*_*c*_*(k)*), (**b**) Three cross-correlation coefficients (*ri*_*ab*_*, ri*_*bc*_*,* and *ri*_*ca*_), (**c**) Durbin-Watson factors (*DWi*_*a*_*, DWi*_*b*_*,* and *DWi*_*c*_), (**d**) Tripping signal, (**e**) Three auto-correlation coefficients (*ri*_*a*_*, ri*_*b*_*,* and *ri*_*c*_), and (**f**) Durbin-Watson factors (*dDWi*_*a1*_*, dDWi*_*b1*_*,* and *dDWi*_*c1*_).

Figure 19 show the results for experiment 11. This investigation is phase *‘A’* loss for the current transformer of the *‘A’* phase. The three-phase secondary currents (*i*_*a*_*, i*_*b*_*,* and *i*_*c*_) are described in Fig. 19a. The three cross-correlation factors (*ri*_*ab*_*, ri*_*bc*_*,* and *ri*_*ca*_) are demonstrated in Fig. 19b, and the three Durbin-Watson factors (*DWi*_*a*_*, DWi*_*b*_*,* and *DWi*_*c*_) are depicted in Fig. 19c. Figure 19d diagrams the tripping signal due to the phase *‘A’* loss during the display time, which indicates the value of + 1.0. Figure 19e offers the three auto-correlation factors (*ri*_*a*_*, ri*_*b*_*,* and *ri*_*c*_), and the three Durbin-Watson factors (*dDWi*_*a1*_*, dDWi*_*b1*_*,* and *dDWi*_*c1*_) are presented in Fig. 19f.

The technique results illustrate the state of phase *‘A’* loss for the transformer. The values of the Durbin-Watson factors (*DWi*_*a*_*,* and *dDWi*_*a1*_) are lower than + 0.5, while the values of the Durbin-Watson factors (*DWi*_*b*_*, DWi*_*c*_*, dDWi*_*b1*_*,* and *dDWi*_*c1*_) are nearly zero during the display time. The values of the auto-correlation factors (*ri*_*b*_ and *ri*_*c*_) factors are almost of + 1.0, while the values of auto-correlation factor (*ri*_*a*_) are within ± 0.5. Also, the values of the three cross-correlation coefficients (*ri*_*ab*_*, ri*_*bc*_*,* and *ri*_*ca*_) are laying within ± 0.5. As a result, the operating points of both cross-correlation and auto-correlation factors are existent within the tripping zones. Thus, the protection scheme based on the Durbin-Watson and correlation algorithms is efficacious in the case of phase *‘A’* loss. The protection method constantly monitors the three-phase currents of the transformer, and emits a tripping signal to the annunciator panel. In this test, the protection tripping time is approximately 34.5 ms.

#### Experiment 12: power transformer shutdown

**Fig. 20:**
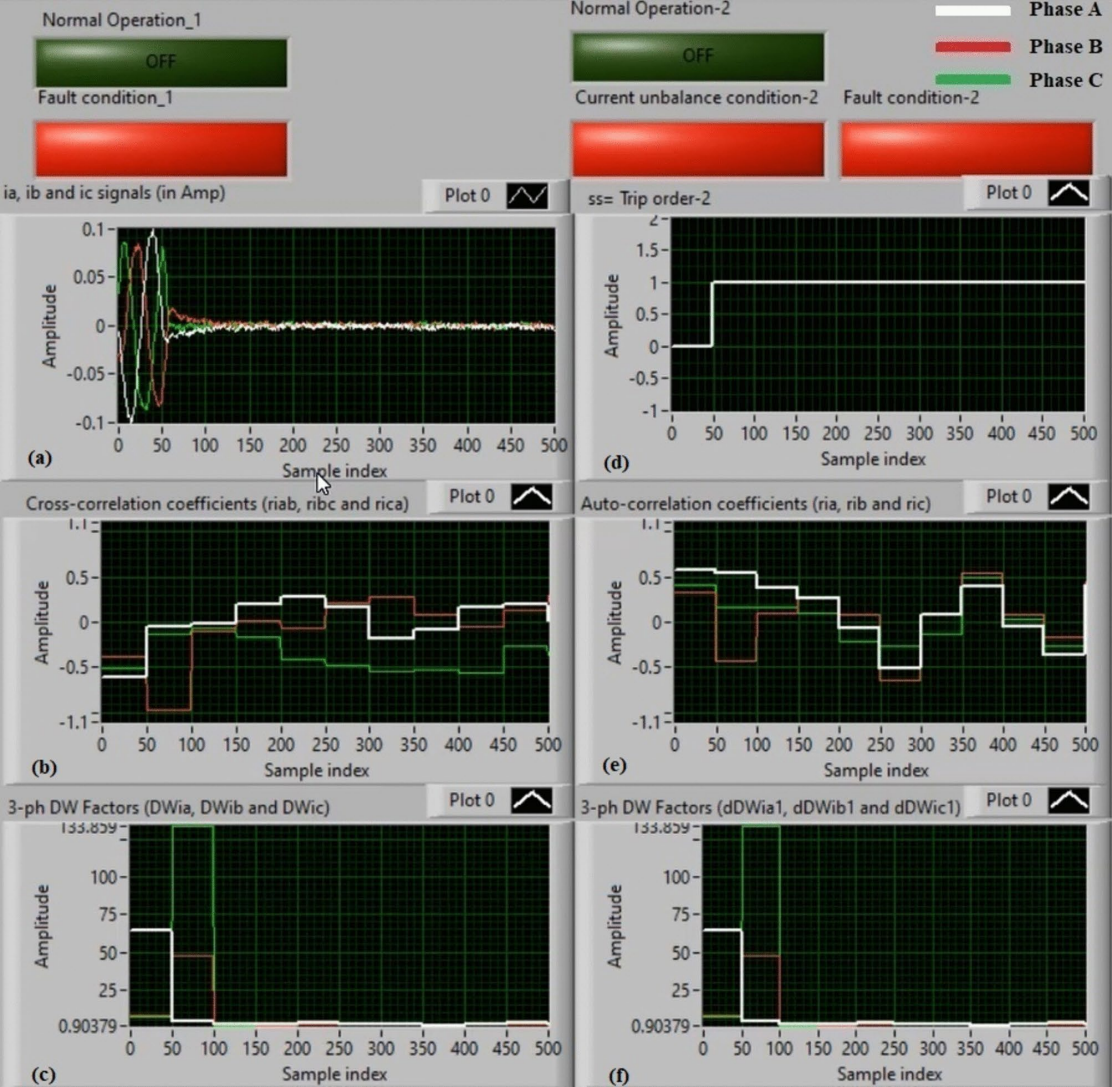
Algorithm results for experiment 12. (**a**) Three-phase currents (*i*_*a*_*(k), i*_*b*_*(k),* and *i*_*c*_*(k)*), (**b**) Three cross-correlation coefficients (*ri*_*ab*_*, ri*_*bc*_*,* and *ri*_*ca*_), (**c**) Durbin-Watson factors (*DWi*_*a*_*, DWi*_*b*_*,* and *DWi*_*c*_), (**d**) Tripping signal, (**e**) Three auto-correlation coefficients (*ri*_*a*_*, ri*_*b*_*,* and *ri*_*c*_), and (**f**) Durbin-Watson factors (*dDWi*_*a1*_*, dDWi*_*b1*_*,* and *dDWi*_*c1*_).

Figure 20 present the results obtained for experiment 12. This test is recorded both before and after the transformer shutdown. The three-phase secondary currents (*i*_*a*_*, i*_*b*_*,* and *i*_*c*_) are illustrated in Fig. 20a. The three cross-correlation estimators (*ri*_*ab*_*, ri*_*bc*_*,* and *ri*_*ca*_) are depicted in Fig. 20b, and the three Durbin-Watson factors (*DWi*_*a*_*, DWi*_*b*_*,* and *DWi*_*c*_) are demonstrated in Fig. 20c. In Fig. 20d, the tripping signal is + 1.0 during the transformer shutdown extent. Figure 20e diagrams the three auto-correlation estimators (*ri*_*a*_*, ri*_*b*_*,* and *ri*_*c*_), and the three Durbin-Watson factors (*dDWi*_*a1*_*, dDWi*_*b1*_*,* and *dDWi*_*c1*_) are shown in Fig. 20f.

As shown in Fig. 20, the algorithm results indicate that the transformer shuts down. The values of the Durbin-Watson factors suddenly increase, exceeding the threshold value (*ΔW* =  + 0.1) during the transformer shutdown. Besides, the operating points of both cross-correlation and auto-correlation coefficients are present within the tripping zones. Consequently, the developed relay based on the Durbin-Watson and correlation techniques works when the transformer is turned off. The protection system monitors the three-phase currents of the transformer continuously, and sends a tripping flag to the annunciator board.

The practical results of the suggested protection scheme are summarized below.

1. It is possible to establish a fast digital protection scheme using the Durbin-Watson and the Pearson similarity functions (as illustrated in all the experiments included in Table 4),
2. Table 4 shows that the Durbin-Watson algorithm is faster than the Pearson similarity algorithm, even though they are dimensionless quantities (as illustrated in all the experiments except experiment 11),
3. A sensitive protection scheme could be used to detect the phase-to-neutral, turn-to-turn, and phase-to-phase faults (as shown in experiments from 1 to 10), as well as light short-circuit currents and imbalanced currents,
4. The Durbin-Watson method is more sensitive at detecting shunt faults than the correlation method (as demonstrated in experiments from 8 to 10),
5. The continuous monitoring of phase loss and equipment shutdown can be demonstrated in experiments 11 and 12, respectively,
6. The Pearson correlation algorithm is considered a redundant protection for the Durbin-Watson algorithm, and vice versa, which can work if one of them fails to trip (as demonstrated in experiments 1 and 7), and
7. New mathematical models, derived using the Durbin-Watson and the Pearson correlation functions, can be employed to specify the convenient tripping time of the protective relay for different fault scenarios (as stated in Eqs. (10) and (12), respectively).

**Table 4:** The tripping times of the protection scheme based on the Durbin-Watson and Pearson functions.

Experiment number	Fault position	The tripping times of the protection scheme based on the Durbin-Watson and Pearson similarity functions (Select *K*_*s1*_ = 1.0, *K*_*s2*_ = 0.1, *DW*_*pu*_ = + 0.1 and *ri*_*pu*_ = + 0.95)					
		Relay action	Max (*DWi*_*a*_, *DWi*_*b*_ or *DWi*_*c*_)	Min (*ri*_*a*_*, ri*_*b*_ or *ri*_*c*_)	*T*_*t1*_ (milli-seconds)	*T*_*t2*_ (milli-seconds)	Actual tripping time Min (*T*_*t1*_ or* T*_*t2*_) (milliseconds)
Experiment 1	Winding-to-neutral fault (C1-N)	Tripping	19.04	+ 1.00	5.25	Infinity	5.25
Experiment 2	Winding-to-neutral fault (C2-N)		1.164	0.00	85.91	105.26	85.91
Experiment 3	Winding-to-neutral fault (C3-N)		115.13	+ 0.20	0.868	160.0	0.868
Experiment 4	Winding-to-neutral fault (B1-N)		1.063	+ 0.15	94.07	143.75	94.07
Experiment 5	Winding-to-neutral fault (B2-N)		85.51	+ 0.25	1.170	178.57	1.170
Experiment 6	Turn-to-turn fault (A1-A2)		19.98	− 0.20	5.000	69.565	5.000
Experiment 7	Turn-to-turn fault (A2-A3)		0.827	+ 0.98	120.92	Infinity	120.92
Experiment 8	Winding-to-winding fault (B1-C1)		49.38	+ 0.15	2.025	143.75	2.025
Experiment 9	Winding-to-winding fault (B2-C2)		109.05	− 0.20	0.917	69.565	0.917
Experiment 10	Winding-to-winding fault (B3-C3)		209.51	− 0.12	0.477	82.243	0.477
Experiment 11	Phase A loss		0.512	− 0.50	195.31	34.483	34.483
Experiment 12	Power transformer shutdown		133.86	− 0.70	0.747	18.181	0.747 (actually, the tripping has been accomplished)

The quantitative findings of the current imbalance coefficients based on the Durbin-Watson and correlation algorithms can be summarized below. Table 5 contains the numerical values of the current imbalance coefficients based on the Durbin-Watson factors for the protection scheme, which can be analyzed as follows:

1. In the case of perfectly balanced currents, the current imbalance coefficients are nearly 0.0%,
2. The three current imbalance coefficients are acceptable when their numerical values are within + 10.0%,
3. The three current unbalance coefficients are unacceptable if their numerical values are greater than + 10.0%,
4. In the instances of turn-to-turn faults, the faulty phase records the maximum coefficient of current unbalance with respect to those of the two healthy phases (as shown in experimental tests 6 and 7),
5. In the instances of winding-to-winding faults, the healthy phase records the maximum coefficient of current unbalance compared to those of the two faulty phases (as shown in experimental tests 8, 9, and 10), and
6. In the case of ‘*A’* phase loss, the current imbalance coefficient of this phase is the highest (as shown in experimental test 11).

**Table 5:** The numerical values of the current imbalance coefficients based on the Durbin-Watson factors.

Experiment number	Fault position	The current imbalance coefficients based on the Durbin-Watson factors for the protection scheme Imbalance coefficients during the fault period *UFi*_*1*_ = The maximum value of (*UFi*_*a1,*_* UFi*_*b1,*_ or* UFi*_*c1*_)								
		Unbalance/balance	DWi_a_	DWi_b_	DWi_c_	DWi_m_	UFi_a1_ (%)	UFi_b1_ (%)	UFi_c1_ (%)	UFi_1_ (%)
Experiment 1	Winding-to-neutral fault (C1-N)	Unacceptable imbalanced current (Tripping action)	5.00	19.04	3.50	9.180	45.54	107.4	61.88	107.4
Experiment 2	Winding-to-neutral fault (C2-N)		0.72	0.90	1.16	0.927	22.30	2.8777	25.18	25.18
Experiment 3	Winding-to-neutral fault (C3-N))		75.0	115.1	25.0	71.7	4.60	60.53	65.13	65.13
Experiment 4	Winding-to-neutral fault (B1-N)		1.06	0.58	0.75	0.797	33.05	27.20	5.86	33.05
Experiment 5	Winding-to-neutral fault (B2-N)		12.0	30.0	85.51	42.5	71.77	29.42	101.2	101.2
Experiment 6	Turn-to-turn fault (A1-A2)		19.98	6.50	6.80	11.09	80.11	41.41	38.71	80.11
Experiment 7	Turn-to-turn fault (A2-A3)		0.827	0.40	0.05	0.426	94.28	6.03	88.25	94.28
Experiment 8	Winding-to-winding fault (B1-C1)		0.00	49.39	47.6	32.33	100.0	52.77	47.23	100.0
Experiment 9	Winding-to-winding fault (B2-C2)		0.00	109.0	109.0	72.67	100.0	50.00	50.00	100.0
Experiment 10	Winding-to-winding fault (B3-C3)		0.00	269.5	175	148.2	100.0	81.88	18.11	100.0
Experiment 11	Phase A loss		0.513	0.00	0.00	0.171	200	100.0	100.0	200
Experiment 12	Power transformer shutdown		65.0	50.0	133.8	82.93	21.62	39.71	61.33	61.33 (Actually, the tripping has been accomplished)

Currents unbalance is acceptable when *UFi*_*1*_ is within *Δu* =  + 10%.

Table 6 includes the numerical values of the current imbalance coefficients based on the correlation factors for the protection scheme. The table manifests the following:

1. In the scenario of perfectly symmetrical currents, the current imbalance coefficients are almost 0.0%,
2. The permissible limit for the three current imbalance coefficients can be set to be + 10.0%,
3. The impermissible limit for the three current imbalance coefficients can be higher than + 10.0%,
4. In the incidence of ‘*A’* phase loss, the current asymmetry coefficient of ‘*A’* phase is the largest (as illustrated in experiment 11),

**Table 6:** The numerical values of the current imbalance coefficients based on the correlation factors for the protection scheme.

Experiment number	Fault position	The current imbalance coefficients based on the Pearson similarity function for the protection scheme Imbalance coefficients during the fault period *UFi*_*2*_ = The maximum value of (*UFi*_*ab2,*_* UFi*_*bc2,*_ or* UFi*_*ca2*_)							
		Unbalance/balance	ri_ab_	ri_bc_	ri_ca_	UFi_ab2_ (%)	UFi_bc2_ (%)	UFi_ca2_ (%)	UFi_2_ (%)
Experiment 1	Winding-to-neutral fault (C1-N)	Unacceptable imbalanced current (Tripping action)	− 1.0	− 1.0	+ 0.8	50	50	130	130
Experiment 2	Winding-to-neutral fault (C2-N)		− 1.0	− 1.0	+ 1.0	50	50	150	150
Experiment 3	Winding-to-neutral fault (C3-N))		− 1.0	− 0.90	+ 1.0	50	40	150	150
Experiment 4	Winding-to-neutral fault (B1-N)		+ 0.80	− 1.0	− 0.95	130	50	45	130
Experiment 5	Winding-to-neutral fault (B2-N)		+ 0.95	− 1.0	− 0.90	145	50	40	145
Experiment 6	Turn-to-turn fault (A1-A2)		− 1.0	+ 0.95	− 1.0	50	145	50	145
Experiment 7	Turn-to-turn fault (A2-A3)		− 0.52	− 0.25	− 0.80	2	25	30	30
Experiment 8	Winding-to-winding fault (B1-C1)		− 0.80	− 1.0	+ 0.75	30	50	125	125
Experiment 9	Winding-to-winding fault (B2-C2)		− 0.90	− 1.0	+ 0.95	40	50	145	145
Experiment 10	Winding-to-winding fault (B3-C3)		− 0.85	− 1.0	+ 0.80	35	50	130	130
Experiment 11	Phase A loss		+ 0.41	− 0.44	− 0.37	91	6	13	91
Experiment 12	Power transformer shutdown		+ 0.30	− 1.0	− 0.10	80	50	40	80 (Actually, the tripping has been accomplished)

Currents unbalance is acceptable when *UFi*_*2*_ is within *Δu* =  + 10%.

## Protection scheme features

The suggested strategy improved with Durbin-Watson and correlation functions represents a significant development in digital protection systems for power transformers. This intelligent scheme not only increases the responsiveness and precision of the fault detection, but also provides high sensitivity to light current levels and inter-turn faults. The two functions within this scheme can be used to analyze and make decisions online, resulting in a more efficient and resilient power network. The laboratory results manifest a considerable enhancement in fault detection sensitivity, speed and accuracy, avoiding the likelihood of power transformer failure and reducing downtime. Furthermore, the ability of the protection system to comprehend and respond to incoming data demonstrates ongoing its optimization and robustness against unforeseen faults in power grids.

### Scheme performance measurement

The protection characteristics of the scheme have been measured over a period of three months. During this period, the total number of correct and incorrect trips in this project was 840.0, out of which 7.0 were incorrect trips. The suggested scheme was unable to trip on 8.0 occasions. The proposal was constrained 75 times with no problems when the experimental system was healthy. The quantitative analysis is pertaining to the characteristics of the protection scheme under the impact of different scenarios of faults and current transducer errors is illustrated in Table 7^41,42^.

**Table 7:** Quantitative analysis for the characteristics of the protection scheme.

Laboratory system instance	Number of scenarios	Malfunction times
The total number of trips (correct + incorrect)	840	7
The total number of normal operations	75	0
Estimation of the characteristics of the protection scheme	Y_1_ = The total number of experiments = 915	Malfunction times = 7
	Y_2_ = The total number of trips	= 840
	Y_3_ = The times of correct trips	= 833
	Y_4_ = The times of tripping failures	= 8
	Y_5_ = The times of desirable trips	= 833 + 8 = 841
	Y_6_ = The times of incorrect trips	= 840 − 833 = 7
	Y_7_ = Y_5_ + Y_6_	= 841 + 7 = 848
	$D= \frac{Y3 }{Y5}\times 100$ %	= $\frac{833}{841}$ $\times$ 100 = $99.05 \%$
	$S= \frac{Y3}{Y2}\times 100 \%$	= $\frac{833}{840}\times$ 100 = $99.17 \%$
	$R= \frac{Y3}{ Y7}\times 100 \%$	= $\frac{833}{848}\times$ 100 = $98.23 \%$
	$A= \frac{Y1-Y4-Y6}{Y1}\times 100 \%$	= $\frac{915-8-7 }{915} \times$ 100 = 98.36%

*D* Dependability, *S* security, *R* reliability, *A* accuracy.

### Protection scheme properties

The main characteristics of the protection strategy for the three-phase transformer can be summarized as follows:

1. Sound and faulty windings of the power transformer can be discerned,
2. In the case of no fault, the Durbin-Watson factor for any phase current vanishes, while its amplitude is considerable in the fault incidence,
3. It can ascertain different faults in the primary/secondary windings of the power transformer, including turn-to-turn, winding-to-neutral, and winding-to-winding faults,
4. The disparity between balanced and unbalanced currents can be achieved,
5. The DW index serves as a loss function without dimension, which can be used to divide the asymmetry in three-phase current waveforms into diverse levels,
6. Swiftly identify the different forms of faults,
7. The proposal is beneficial because it can be used in practice,
8. It can detect the onset of faults immediately,
9. It is accurate and reliable,
10. The advanced approach can be used to monitor online the three-phase currents of other electrical AC machines,
11. The developed approach can be applied to smart grids with a variety of power and voltage scales,
12. The protection properties can be customized utilizing the protection pre-setting values and the data package size,
13. The method is inactive when there is no fault, while it becomes active in the instances of fault and unbalanced currents,
14. The advanced method is stable when varying the parameter specifications of power transformers and current transducers,
15. The protection scheme can work with other digital processes/systems, such as digital protection devices, fault recorders, and event recorders, to make it more reliable and redundant,
16. The protection method only requires the local current measurements, speeding up the process of data transfer and processing,
17. The method can be applied to single-phase or three-phase windings of power transformers,
18. The algorithm exhibits robustness to a variety of fault types, fault time periods, fault inception times, and fault resistances,
19. No estimation of protection settings is needed,
20. The data set and the settings of Durbin-Watson and correlation can be modified to tune the fault detection sensitivity. To make the algorithm more sensitive, the predetermined settings and the data set should be reduced, and
21. The approach can be used to protect the windings of different AC machines, such as power transformers, induction machines, and synchronous machines, along with other power system components.

### Comparison

Table 8 compares the suggested Durbin-Watson and correlation-based protection scheme with other numerical protection schemes (such as differential current relay, REF relay, current imbalance relay, phase overcurrent relay). The table indicates that the Durbin-Watson and correlation-based protection scheme is more efficient and efficacious.

**Table 8:** Comparison of the suggested protection scheme with other numerical protection schemes.

Sr. N	Protection attributes	Suggested protection scheme	Differential current relay	REF relay	Current imbalance relay	Phase overcurrent relay
NA = Not Applicable, A = Applicable, H = Higher, L = Lower, Y = Yes, N = No, and RMS = Root Mean Square						
1	Data Set/RMS concept	Data Set (customized)	RMS	RMS	RMS	RMS
2	Symmetrical faults detection	A	A	NA	NA	A
3	Currents imbalance detection	A	A	A	A	NA
4	Data of equipment parameters	NA	A	A	A	A
5	Required number of CTs	3	6	4	3	3 or 4
6	Protection sensitivity	H	L	H	L	L
7	Protection Speed	H (customized)	H	H	Based on tripping characteristics	Based on tripping characteristics
8	Protection adaptability	A	NA	NA	NA	NA
9	Protection accuracy	H	H	H	H	H
10	Protection reliability	H	H	H	H	H
11	Detection of diverse faults					
	Symmetrical faults	Y	Y	N	N	Y
	Unsymmetrical faults	Y	Y	Y	Y	Y
	Inter-turn faults	Y	N	N	Y	N
	Phase faults	Y	Y	N	Y	Y
	Grounding faults	Y	Y	Y	Y	N

## Contributions

Below, the primary contributions can be enumerated.

1. The Durbin-Watson algorithm can be amalgamated with the correlation algorithm to create a new digital system for protecting the primary/secondary windings of the power transformer. Accordingly, it can also be used to protect the three-phase windings of other AC machines,
2. A new set of tripping characteristic curves based on the Durbin-Watson function and another tripping characteristic curves based on the correlation function have been presented, which can be used to determine the suitable tripping time that corresponds to the disturbance level in the power quality of the fault currents,
3. A new mathematical expression based on the Durbin-Watson indices and another mathematical expression based on the correlation indices can be used to measure current imbalance coefficients for power transformers. These expressions can be also applied to estimate the voltage imbalance coefficients for diverse types of AC machines, and
4. Several tools are available to control the protection sensitivity and speed for both the Durbin-Watson and correlation algorithms.

## Conclusions

A smart protection strategy based on the Durbin-Watson and similarity algorithms for power transformers protection has been presented in this paper. The DAC has received the secondary currents from the current transducers built to protect the power transformer, which has been used to turn analog signals into discrete values. After that, the Durbin-Watson and Pearson correlation algorithms have been processed with the LABVIEW application program. Comprehensive experimental tests have been conducted on the power transformer with tapped windings in order to examine the accuracy and efficiency rates of the protection system under different fault scenarios. For each scenario, the three-phase secondary current signals, the three cross-correlation coefficients, the three auto-correlation coefficients, and six Durbin-Watson factors have been recorded for statistical analysis. The practical results demonstrate the effectiveness of the protection strategy, where its security and dependability levels overtake 99%, and its accuracy and reliability levels override 98%. Additionally, two models of tripping curves have been developed, one employing the Durbin-Watson indices and the other employing the auto-correlation indices. These models have allowed the protection scheme to determine the appropriate tripping times for different fault forms on the power transformer windings, such as winding-to-winding, winding-to-neutral, and turn-to-turn faults. In summary, the suggested protection scheme based on the Durbin-Watson and Pearson similarity functions has the ability to:

1. Specify the instance of faults on the power transformer windings, including but not limited to turn-to-turn, winding-to-neutral and winding-to-winding faults. Additionally, it is able to monitor the circumstances of phase loss and equipment shutdown,
2. Discern between the balance and unbalance of the currents,
3. Measure the asymmetry size of the power transformer currents,
4. Value the extent of the disturbance in each phase current, and
5. Figure the optimum tripping time for the protection based on the Durbin-Watson and correlation indices for the fault currents.

The proposed approach behaves like a backup protection for the differential current protection that serves as a primary protection for the power transformer. Thus, the analysis of harmonic contents in advanced differential current relays for the power transformers protection should be used to discriminate between internal faults, inrush currents and over-fluxing instances. Nevertheless, the protection mechanism can satisfy the functional tasks of the differential protection when six current transformers are constructed at the two ends of the three-phase primary/secondary windings of the power transformer. This notion will be applied practically in the future.

## Supplementary Information

Supplementary Information.

## Data Availability

All data generated or analysed during this study are included in this published article [and its supplementary information files].
