# Supplementary material for: Digital protection scheme based on Durbin Watson and Pearson similarity indices for current signals practically applied to power transformers
Source: Sci Rep. 2025 Apr 10;15:12214. doi: 10.1038/s41598-025-91491-1 (PMC11985498; doi:10.1038/s41598-025-91491-1)
Supplement: Supplementary file 1 — Supplementary Information. [file 41598_2025_91491_MOESM1_ESM.pdf]

Nomenclatures:

Symbols	Abbreviations	Symbols	Abbreviations
3LNF	Three Line-to-Neutral Fault,	*S* or *X*	The subscript *S* or *X* stands for phase *A*, *B* or *C*.
CT	Current Transformer,	DWi_m	It is the average of summation of the three Durbin-Watson factors (DWi_a, DWi_b and DWi_c),
CTR	Current Transformer Ratio,	DW_{pu}	Durbin-Watson pickup value (the chosen value is DW_{pu}= + 0.10),
CB	Circuit Breaker,	DWi_s	Durbin-Watson factor estimated between each two data sets differing by a single cycle,
DAC	Data Acquisition Card,	ri_{sx}	The cross-correlation coefficient estimated between two corresponding data packages for the two phase currents ($i_s(k)$ and $i_x(k)$) acquired for *S* and *X* phases of the power transformer windings,
DLF	Double Line Fault,	ri_{ab}, ri_{bc}, and ri_{ca}	The three cross-correlation coefficients calculated between each two different phase currents of the three phase currents ($i_a(k)$, $i_b(k)$ and $i_c(k)$),
DLNF	Double Line-to-Neutral Fault	ri_s	The auto-correlation coefficient computed between two data packages differing by a single cycle for the phase current $i_s(k)$,
SLNF	Single-Line-to-Neutral Fault,	ri_{sx}	The cross-correlation coefficient estimated between two corresponding data packages for the two phase currents ($i_s(k)$ and $i_x(k)$) acquired for *S* and *X* phases of the power transformer windings,
$i_a(k)$, $i_b(k)$ and $i_c(k)$	The numerical values of the three-phase currents at position '*k*' measured at the supply end of the power transformer windings,	ri_a, ri_b, and ri_c	The three auto-correlation coefficients calculated for the three phase currents ($i_a(k)$, $i_b(k)$ and $i_c(k)$), respectively,
$i_a(k-N_s)$	The sample value of the current (i_a) at the sample index ($k-N_s$),	DWi_a, DWi_b, and DWi_c	The three DW factors computed using mathematical equation (1),
$i_b(k-N_s)$	The sample value of the current (i_b) at the sample index ($k-N_s$),	$dDWi_{a1}$, $dDWi_{b1}$, and $dDWi_{c1}$	The three DW factors computed using mathematical equation (2),
$i_c(k-N_s)$	The sample value of the current (i_c) at the sample index ($k-N_s$),	T_{s1}	The estimated tripping time (in milliseconds) of the protection for '*S*' phase using the Durbin-Watson factor (DWi_s),
$i_s(k)$	The measured current sample taken at a position '*k*' for the *S* phase,	T_{a1}	The estimated tripping time (in milliseconds) of the protection for '*A*' phase using the Durbin-Watson factor (DWi_a),
$i_s(k-N_s)$	The measured current sample taken at a position '$k-N_s$' for the *S* phase,	T_{b1}	The estimated tripping time (in milliseconds) of the protection for '*B*' phase using the Durbin-Watson factor (DWi_b),
$i_x(k)$	The current measurement at the position (*k*) measured for the *X* phase at the supply side of the power transformer windings,	T_{c1}	The estimated tripping time (in milliseconds) of the protection for '*C*' phase using the Durbin-Watson factor (DWi_c),

$i_x(k\text{-}N_s)$	The current measurement at the position '$k\text{-}N_s$' measured for the X phase at the supply side of the power transformer windings,	T_{t1}	The actual tripping time (in milliseconds) of the relay; it is the lowest value of (T_{a1}, T_{b1}, or T_{c1}),
UFi_{a1}	The unbalance coefficient derived using the Durbin-Watson factor (DWi_a),	K_{s1}	The chosen value of time multiplier using the Durbin-Watson algorithm,
UFi_{b1}	The unbalance coefficient derived using the Durbin-Watson factor (DWi_b),	T_{s2}	The estimated tripping time (in milliseconds) of the protection for 'S' phase using the auto-correlation coefficients (ri_s),
UFi_{c1}	The unbalance coefficient derived using the Durbin-Watson factor (DWi_c),	T_{a2}	The estimated tripping time (in milliseconds) of the protection for 'A' phase using the auto-correlation coefficients (ri_a),
UFi_1	It is the maximum value of three-phase unbalance coefficients (UFi_a, UFi_b or UFi_c) based on Durbin-Watson factors (DWi_a, DWi_b,, DWi_c),	T_{b2}	The estimated tripping time (in milliseconds) of the protection for 'B' phase using the auto-correlation coefficients (ri_b),
UFi_{ab2}	The unbalance coefficient derived using the cross-correlation coefficient (ri_{ab}),	T_{c2}	The estimated tripping time (in milliseconds) of the protection for 'C' phase using the auto-correlation coefficients (ri_c),
UFi_{bc2}	The unbalance coefficient derived using the cross-correlation coefficient (ri_{bc}),	T_{t2}	The actual tripping time (in milliseconds) of the relay; it is the minimum quantity of (T_{a2}, T_{b2}, or T_{c2}),
UFi_{ca2}	The unbalance coefficient derived using the cross-correlation coefficient (ri_{ca}),	ri_{pu}	The prescribed auto-correlation pickup of the relay (it is $ri_{pu} = 1.0 - \Delta r_2$),
UFi_2	The imbalance coefficient (in %) based on the cross-correlation factors (ri_{ab}, ri_{bc}, and ri_{ca}) calculated for the three-phase transformer currents; it is the maximum value of the three-phase coefficients (UFi_{ab2}, UFi_{bc2}, or UFi_{ca2}) based on the three cross-correlation estimators (ri_{ab}, ri_{bc}, and ri_{ca}), respectively.	K_{s2}	The chosen value of time multiplier using the auto-correlation algorithm,
N_s	The number of measurements per a single cycle for phase current,	Δr_1	The cross-correlation deviation value used in the protection algorithm; it is selected +0.10,
N_w	The number of measurements per the data set area ($N_w \leq N_s$) of the phase current,	Δr_2	The auto-correlation deviation value used in the protection algorithm; it is selected +0.05,
DWi_a	The Durbin-Watson factor computed for $i_a(k)$ of the phase 'A' primary winding of power transformer,	ΔW	The pickup value of the Durbin-Watson factor used in the protection algorithm; it is selected +0.10,
DWi_b	The Durbin-Watson factor computed for $i_b(k)$ of the phase 'B' primary winding of power transformer,	Δu	The unbalance factor deviation; it is selected +0.10,
DWi_c	The Durbin-Watson factor computed for $i_c(k)$ of the phase 'C' primary winding of power transformer,	$Snsv_1$	The sensitivity of the Durbin-Watson algorithm,
Acc_1	The accuracy of the Durbin-Watson algorithm,	$Snsv_2$	The sensitivity of the correlation algorithm,
Acc_2	The accuracy of the correlation algorithm,		

Appendix 1: The parameters' data of the power model components

The parameter of the power model components	Data
**<u>Three-phase power supply:</u>**	
Rated line voltage	*380 V*
Rated frequency	*50 Hz*
**<u>Three phase auto-transformer (under test):</u>**	
Each phase winding includes 10 Taps	*22 V between each two-taps of the same phase*
Rated power	*4 kVA (Star connection)*
Rated line voltage	*400 V*
Nominal frequency	*50 Hz*
Rated line current	*6 A*
**<u>Three-phase induction motor (as a load):</u>**	
Rated power	*2.2 kW (Star connection), or 3 HP*
Rated line voltage	*380 V*
Nominal frequency	*50 Hz*
Rated line current	*4.9 A*
Rated speed	*2840 rpm*
Pf	*0.8*
**<u>Current transformers (CTs):</u>**	
Current Transformer turns' Ratio (CTR)	*200/5*
Frequency	*47…50…63 Hz*
CT accuracy class	*1.0*
Rated burden	*2.5 VA*
CT burden	*1 Ω*
**<u>Miniature Circuit Breaker (MCB1)</u>**	
Phase type	Three phase
Rated current	63 A
Rated voltage	400 V

Appendix 2: Input quantities for the protection algorithm

**Quantity designation**	**Quantity description**	**Numerical value**
$i_a(n)$, $i_b(n)$ **and** $i_c(n)$	The current measurements of *a*, *b* and *c* phases, respectively, at the instant *n* taken at the supply end of the power transformer windings,	The measurements are taken online
F_c	The fundamental cycle frequency for electrical signals	50 *Hz*
T_c	The cycle time interval	20 milliseconds
F_{sp}	The frequency rate of the digital system	2.5 *kHz*
T_{sp}	The sampling time interval	0.4 milliseconds
N_s	The number of measurements per a single cycle for phase current,	50 samples/data set
N_w	The number of measurements per the data set area of the phase current, ($N_w \leq N_s$)	50 samples/cycle
T_{ds}	The full display time	10 cycles
Δr_1	The cross-correlation deviation value used in the protection algorithm,	+0.10
Δr_2	The auto-correlation deviation value used in the protection algorithm,	+0.05
ΔW	The pickup value of the Durbin Watson factor used in the protection algorithm,	+0.10
Δu	The unbalance factor deviation,	+0.10
